# High-Resolution Mapping and Dynamics of the Transcriptome, Transcription Factors, and Transcription Co-Factor Networks in Classically and Alternatively Activated Macrophages

**DOI:** 10.3389/fimmu.2018.00022

**Published:** 2018-01-18

**Authors:** Amitabh Das, Chul-Su Yang, Sarder Arifuzzaman, Sojin Kim, Sun Young Kim, Kyoung Hwa Jung, Young Seek Lee, Young Gyu Chai

**Affiliations:** ^1^Institute of Natural Science and Technology, Hanyang University, Ansan, South Korea; ^2^Department of Molecular and Life Sciences, Hanyang University, Ansan, South Korea; ^3^Department of Bionanotechnology, Hanyang University, Seoul, South Korea

**Keywords:** macrophages, transcription factors, interferon-g, RNA-sequencing, lipopolysaccharide

## Abstract

Macrophages are the prime innate immune cells of the inflammatory response, and the combination of multiple signaling inputs derived from the recognition of host factors [e.g., interferon-g (IFN-γ)] and invading pathogen products (e.g., toll-like receptors (TLRs) agonists) are required to maintain essential macrophage function. The profound effects on biological outcomes of inflammation associated with IFN-γ pretreatment (“priming”) and TLR4 ligand bacterial lipopolysaccharide (LPS)-induced macrophage activation (M1 or classical activation) have long been recognized, but the underlying mechanisms are not well defined. Therefore, we analyzed gene expression profiles of macrophages and identified genes, transcription factors (TFs), and transcription co-factors (TcoFs) that are uniquely or highly expressed in IFN-γ-mediated TLR4 ligand LPS-inducible versus only TLR4 ligand LPS-inducible primary macrophages. This macrophage gene expression has not been observed in macrophage cell lines. We also showed that interleukin (IL)-4 and IL-13 (M2 or alternative activation) elicited the induction of a distinct subset of genes related to M2 macrophage polarization. Importantly, this macrophage gene expression was also associated with promoter conservation. In particular, our approach revealed novel roles for the TFs and TcoFs in response to inflammation. We believe that the systematic approach presented herein is an important framework to better understand the transcriptional machinery of different macrophage subtypes.

## Introduction

Macrophages are major innate immune cell populations that respond to a microbial insult or danger signal, are specialized for phagocytosis, and play a central role in the immune response (1, 2). Most tissue-resident macrophages arise from embryonic origin and have the capacity to self-renewing adult bone marrow-derived macrophages (BMDMs) (2, 3). Their response induces substantial inflammatory responses tailored mainly by gene-specific transcriptional regulation, which could be classically activated, type I subset (M1) and the alternatively activated macrophage type II subset (M2) (4), although recent studies have suggested an avoidance of the complexity of the M1 or M2 subset (5).

Type I subset activity can be polarized by inflammatory stimuli such as lipopolysaccharide (LPS), which, in combination with interferon-g (IFN-γ), induces expression of proinflammatory mediators, such as cytokines, oxidative metabolites, and proteases that play roles in the tissue-destructive pathology associated with inflammatory disease. In contrast, M2 activation is induced by the stimulation of interleukin (IL)-4 and*/*or IL-13, which may be engaged in processes, such as wound repair and support type 2-mediated disease, homeostasis and remodeling, as well as induces anti-inflammatory cytokines (5, 6). One of the most important endogenous mediators of inflammation of macrophages is the IFN-γ that plays a critical role in promoting macrophage activation, host defense, and immunoediting (7). IFN-γ can sensitize macrophages activation following pathogen products, including LPS challenge (8, 9). In parallel, IFN-γ with toll-like receptors (TLRs) can either augment or suppress the expression of genes related to inflammation (10–12). The interaction of LPS and IFN-γ triggers downstream signaling leading to an inflammatory response. TLR4 recognizes bacterial LPS and induces the mitogen-activated protein kinase (MAPK) pathway, which facilitates activation of the key transcription factors (TFs), nuclear factor κB (NF-κB), and activator protein-1 (AP-1) (13). IFN-γ, in contrast, activates the Janus kinase (JAK) and signal transducer and activator of transcription (STAT) pathway and the subsequent autocrine activities of IFN-γ (10). In addition, STAT1 targeted interferon regulatory factor-1 (IRF1) has also been shown to contribute to a multi-layer integration of IFN-γ signaling (14). Although these downstream signal transduction cascade and their molecules have been discovered in macrophages, the molecular mechanisms of the IFN-γ-mediated TLR4 cross-regulation in murine macrophages remain largely unaddressed and are under active investigation.

It is well-recognized that IL-4 and IL-13 are derived by activated T helper 2 (Th2)-polarized T cells, granulocytes and monocytes/macrophages, as well as new innate immune cell populations, including natural killer T (NKT) cells and innate lymphoid type 2 cells (iLC2). These cytokines are critical for Th2 T cell differentiation and M2 macrophage polarization, as well as for the promotion of allergic responses (15, 16). However, substantial evidence has indicated that the two cytokines mediate the unique physiological functions, with IL-4 primarily involved in Th2 cells differentiation and proliferation, whereas IL-13 playing an important role in effector activities, such as the regulation of airway hypersensitivity, collagen production, and mucus hypersecretion (17, 18). IL-4 and IL-13 are unique in that these cytokines can bind to two distinct receptor complexes; in particular, IL-4 binds to the IL-4Rα chain and the type II receptor. In contrast, IL-13 does not appear to bind IL-4Rα directly, but binding to the IL-13Rα1 chain and can only be activated by type II IL-4R (19). Both pathways facilitate the transcription and stabilization of a subset of genes associated with M2 macrophage polarization (20). Although these downstream signal transduction cascade their molecules have been discovered in macrophages, the molecular mechanisms of the IL-4 and IL-13-mediated functional responses in murine macrophages remain largely unaddressed. Because therapeutic applications targeting IL-4 and IL-13 or their downstream signaling molecules are presently under development aiming to eradicate allergy and asthma, understanding about their molecular mechanisms of action, and contribute to allergic asthmatic inflammation awaits further elucidation.

To date, several genome-scale studies of the transcriptional reprogramming of macrophage polarization, induced by environmental stimuli such as LPS have been conducted to determine comprehensive signatures in macrophages (21–26). However, thus far, a genome-wide search for IFN-γ-mediated TLR4 cross-regulation has not been performed in murine macrophages using massively parallel cDNA sequencing (RNA-sequencing). Thus, we performed RNA-sequencing for gene expression profiling of LPS-stimulated, IFN-γ-primed LPS-stimulated, and IL-4/IL-13-primed BMDMs, thus paving the way for an unbiased digital readout and improved detection at the extremes of the transcriptome of any mammalian cell (27). The outcomes of these studies allowed us to identify a macrophage transcriptional signature for IFN-γ-mediated TLR4 cross-regulation and IL-4/IL-13-primed macrophages. We also identified a unique macrophage transcriptional signature distinguishing them from the RAW264.7 macrophage cell line. Furthermore, we identified *trans* (e.g., altered TF expression, activation, or motif specificity) regulatory elements that may drive distinct gene expression in IFN-γ-primed LPS and IL-4/IL-13-primed macrophages. Altogether, our data provide new perspectives for the biology of different macrophage subtypes.

## Materials and Methods

### RAW 264.7 Macrophage Cell Line

The mouse macrophage cell line, RAW 264.7, was obtained from the American Type Culture Collection (Manassas, VA, USA), and the cells were grown in RPMI medium supplemented with FBS, 100 IU/ml penicillin, and 10 µg/ml of streptomycin (Invitrogen, USA). The cells were maintained in a humidified incubator with 95% air and 5% CO_2_ atmosphere at 37°C. RAW264.7 macrophage cell line were incubated with LPS (100 ng/ml) for the specified times under normal culture conditions. The medium, containing the appropriate agents, was replaced every other day. LPS (L6529; strain 055:B5) were purchased from Sigma-Aldrich, St. Louis, MO, USA.

### Preparation of BMDMs

C57BL/6 mice were purchased from Samtako Bio Korea (Gyeonggi-do, Korea), and the mice were maintained under specific-pathogen-free conditions. BMDMs were isolated from C57BL/6 mice as previously described (28). To initiate differentiation, the medium was supplemented with 25 ng/ml recombinant macrophage colony-stimulating factor (M-CSF) (R&D Systems 416-ML) for 4 days. BMDMs were grown in Dulbecco’s Modified Eagle’s Medium (DMEM; Life Technologies, Carlsbad, CA, USA) that was supplemented with 10% fetal bovine serum (FBS) and 4 mM glutamine (Life Technologies, Carlsbad, CA, USA). The cells were maintained in a humidified incubator with a 95% air, 5% CO_2_ atmosphere at 37°C.

### Polarization of BMDM Macrophages into M1 or M2 Phenotype

Type I subset and M2 macrophages were polarized as described previously (29). Primary BMDM macrophages were polarized toward the M1 phenotype with recombinant IFN-γ (100 U/ml, R&D Systems 485-ML) and/or LPS (100 ng/ml, L6529; strain 055:B5; Sigma-Aldrich, St. Louis, MO, USA) or toward the M2 phenotype with recombinant IL-4 (R&D Systems 404-ML) or recombinant IL-13 (R&D Systems 413-ML) (10 ng/ml) for the specified times under normal culture conditions. Unpolarized cells (M0) served as controls. BMDMs were washed with PBS and harvested for total RNA isolation.

### Total RNA Isolation and cDNA Library Preparation for Transcriptome Sequencing (RNA-seq)

Total RNA was extracted using RNAiso Plus (Takara Bio Inc., Shiga, Japan) and a QIAGEN RNeasy^®^ Mini kit (QIAGEN, Hilden, Germany). BMDMs or RAW264.7 macrophage cell line was completely lysed using RNAiso Plus and then 200 µl of chloroform was added. The tubes were then inverted for 5 min. The mixture was centrifuged at 12,000 × *g* for 15 min at 4°C, and the upper phase was placed into a new tube. A 600 µl volume of 70% ethanol was added and the mixture was applied to an RNeasy mini column. The column was washed with wash buffer. To elute the RNA, RNase-free water (30 µl) was added directly onto the RNase mini column, which was then centrifuged at 12,000 × *g* for 3 min at 4°C. To deplete ribosomal RNA (rRNA) from the total RNA preparations, a RiboMinus Eukaryote kit (Life Technologies, Carlsbad, CA, USA) was used according to the manufacturer’s instructions. RNA libraries were created using a NEBNext^®^ Ultra™ directional RNA library preparation kit for Illumina^®^ (New England Biolabs, Ipswich, MA, USA). The obtained rRNA-depleted total RNA was fragmented into small pieces using divalent cations at elevated temperatures. First-strand cDNA was synthesized using reverse transcriptase and random primers and second-strand cDNA synthesis was then performed using DNA polymerase I and RNase H. The cDNA fragments were processed using an end-repair reaction after the addition of a single “A” base, followed by adapter ligation. These products were purified and amplified using PCR to generate the final cDNA library. The cDNA fragments were sequenced using an Illumina HiSeq2000. Biological triplicate RNA-sequencing for each condition were performed on independent RNA samples from the IFN-γ primed LPS-stimulated and IL-4/IL-13-stimulated BMDMs: control BMDMs (3 samples); IFN-γ primed LPS 1 h (3 samples), IFN-γ primed LPS 4 h (3 samples), IFN-γ primed LPS 12 h (3 samples), IFN-γ primed LPS 24 h (3 samples), IL-4 12 h stimulated (3 samples), IL-13 12 h stimulated (3 samples), and only LPS 4 h (2 samples) and RAW264.7 macrophage cell line: control 4 h (2 samples) and LPS 4 h (2 samples).

### Differentially Expressed Genes (DEGs) Analysis Using RNA-seq Data

FASTQ files from RNA-seq experiments were clipped, trimmed of adapters, and the low-quality reads were removed by the Trimmomatic (30). Quality controlled FASTQ files were aligned to *Mus musculus* UCSC mm10 reference genome sequence using the STAR (version 2.5.1) aligner software (31). To measure differential gene expression, DESeq2 (32) with the default parameters was used. The RNA-seq experiments were visualized using HOMER (33) after custom tracks were prepared for the UCSC Genome Browser.^1^

### Quantitative Real-time Polymerase Chain Reaction (qRT-PCR)

The reverse transcription of the RNA samples was performed as previously described (34). qRT-PCT was performed using an ABI-7500 Real-time PCR System (Applied Biosystems, Waltham, MA, USA) with SYBR Premix Ex-Taq II (Takara Bio Inc., Shiga, Japan) according to the manufacturer’s instructions. The reactions were performed in a total volume of 20 µl that contained 0.4 mM of each primer (Table S1 in Supplementary Material). Each PCR series included a no-template control that contained water instead of cDNA and a reverse transcriptase-negative control for each gene. Every sample was measured in triplicate, and relative quantification was effected by means of the comparative CT (ΔΔCT) method. glyceraldehyde-3-phosphate dehydrogenase (GAPDH) was used a housekeeping gene to normalize the expression data.

### Chromatin Immunoprecipitation (ChIP) Assay

Chromatin Immunoprecipitation experiments were performed as previously described (35). Briefly, chromatin from 1 × 10^7^ cells was used for each immunoprecipitation. The RAW 264.7 macrophage cell line was immunoprecipitated with antibodies against IRF1 (Santa Cruz sc-514544), STAT1 (Santa Cruz sc-345), and normal rabbit IgG (Santa Cruz sc-2025) used as a control. The immunoprecipitated DNA was analyzed by real-time quantitative PCR, with one modification: the cDNA was replaced with immunoprecipitated DNA. The relative enrichment levels indicate the fold changes over the IgG control. Primers used for ChIP-PCR are listed in Table S2 in Supplementary Material.

### Functional Annotation Analysis and Heat Map Construction

To functionally annotate the most significant genes, Gene Set Enrichment Analysis (GSEA) was performed by using the Molecular Signatures Database (MSigDB), version 6.1.^2^ Gene ontology (GO) was analyzed using a modified Fisher’s exact *P*-value in the GSEA program. *P*-values less than 0.001 were considered greatly enriched in the annotation category. We constructed heat maps to view the relative expression patterns of our array data using Multi experiment Viewer (MeV) program (36, 37).

### Upstream Regulator Analysis of Datasets

An Ingenuity Pathway Analysis (IPA) (Ingenuity Systems, CA)^3^ was performed to analyze the most significant canonical pathways and upstream regulator analysis in the datasets as previously described (38). After uploading the datasets, upstream regulator analysis was used to predict the upstream transcriptional regulators on the literature and compiled in the Ingenuity Pathways Knowledge Base (IPAKB). Gene networks were algorithmically generated based on connectivity. The analysis examines how many known targets of the upstream regulators are present in the dataset and also the direction of change. The graphical representation of molecular relationships between upstream regulator and gene products was based on the biological relationship between two nodes was represented as an edge (line). All edges were supported by at least one reference from the literature, textbook, or canonical information in IPAKB. The intensity of node color represented the degree of upregulation (red). The nodes were displayed using shapes to represent functional classes of gene products.

### TF Binding Motif Enrichment Analysis

NCBI reference sequence mRNA accession numbers were subjected to TF binding motif analysis using the web-based software Pscan (39). The JASPAR (40) database of TF binding sequences were analyzed using enriched groups of −950 base pair (bp) sequences to +50 bp of the 5′ upstream promoters. The range −950 to +50 was selected from the range options in Pscan to obtain the best coverage for a −1,000 to +50 bp range. We next used position weight matrices (PWMs) models of TFBSs contained in the TRANSFAC professional database (41). The enrichment of motifs within the target set of macrophage promoters was calculated relative to the frequency of motif occurrence in the mouse genome.

### Statistical Analysis

The data were analyzed using Origin Pro 8 software (Origin Lab Corporation, Northampton, MA, USA). Each value is expressed as the mean ± SEM. All qRT-PCR data were analyzed with SPSS 17.0 software (SPSS Inc., Chicago, IL, USA). The data were tested using one-way ANOVA followed by Tukey’s HSD *post hoc* test. *P < 0.01 and **P < 0.001 were considered significant.

## Results

### LPS Dynamically Regulates Transcriptional Programs in BMDMs

**Figure 1:**
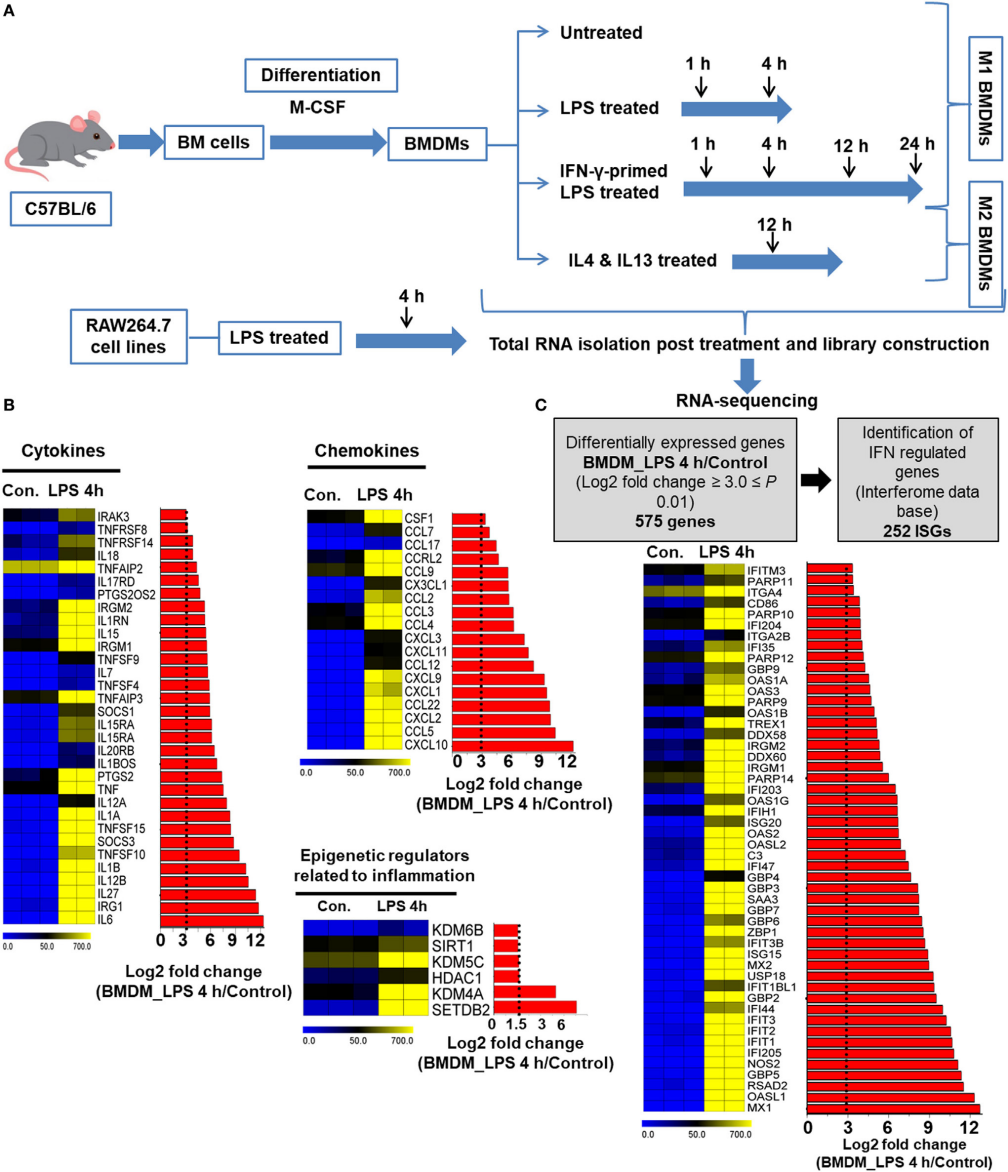
RNA-seq analyses reveal the dynamics of lipopolysaccharide (LPS)-triggered inflammatory response-related genes of bone marrow-derived macrophages (BMDMs). **(A)** Strategy for the RNA-seq experimental steps. **(B)** Heat map representation depicting the expression of positive regulators of inflammatory genes (cytokines, chemokines, and epigenetic regulators) in BMDMs at 4 h after LPS stimulation (*P* ≤ 0.01 and log_2_-fold change ≥ 3.0). BMDMs are compared with the control. **(C)** Identification of interferon regulated genes (IRGs) and an overview of expressed IRGs (*P* ≤ 0.01 and log_2_-fold change ≥ 3.0) in BMDMs compared with the control. Each row shows the relative expression level of a single gene and each column shows the expression level of a single sample. Heat maps were generated with multi experiment viewer (version 4.8) software.

First, we generated *ex vivo* BMDMs from mouse bone marrow immature precursor cells by stimulation with M-CSF that lead to differentiation of monocytes into mature macrophages, as emphasized in a previous report (5). To determine the gene expression pattern in response to LPS stimulation, we used RNA-seq after depletion of rRNA and analyzed the global gene expression patterns in LPS-stimulated and control BMDMs. A detailed outline of the experiments is depicted in Figure 1A. Of note, using qRT-PCR, we observed that transient upregulation of key inflammatory genes, maximal or near-maximal at the 4 h time point (data not shown). Based on these results, we used the 4 h as well as the 1 h time points for gene expression profiling; these time points were also reported in another study (42) determining the general gene expression patterns of macrophage activation by LPS. Using a false discovery rate (FDR ≤ 0.01), *P* ≤ 0.01 and log_2_-fold change ≥1.5 as the cutoff values for the up or downregulation of genes, we identified genes that were altered in LPS-stimulated BMDMs: 596 genes at 1 h and 2,248 genes at 4 h were differentially expressed. Of these, 414 and 1,326 genes were upregulated, and 182 and 922 genes were downregulated at 1 and 4 h, respectively (Figure S1A in Supplementary Material). Among the 414 and 1,326 upregulated genes, 29 and 43% were induced by at least a 3-fold log_2_ change at 1 and 4 h, respectively (Figure S1B in Supplementary Material). Importantly, of the most highly induced genes, 29 and 43% encoded key cytokines, and chemokines were induced by a >3-fold log_2_ change (Figure 1B). To further assess the genes that showed changes in expression >3-fold log_2_ in LPS-treated BMDMs compared with control cells among known (IFN)-regulated genes (IRGs) (43), 252 (44%) IRGs were found to be induced at 4 h (Figure 1C). In addition, our RNA-seq data also identified the induction of gene families implicated in epigenetic regulation. Studies of epigenetic regulation to play an important role in modulating inflammatory genes have recently reported (44). RNA-seq approaches herein identified among multiple regulators of epigenetic families, only histone methyltransferases (SETDB2), histone demethylases (KDM4A), and histone deacetylases (HDAC1) were significantly differentially expressed (Figure 1B). These findings suggest that SETDB2, KDM4A, and HDAC1 have an important role in the regulation of BMDM activation. Taken together, these findings led us to hypothesize that in addition to regulating cytokines and chemokines, LPS may also be a positive regulator of IRGs and epigenetic regulators.

### Time Series, IFN-γ-Primed LPS-Inducible and Only LPS-Inducible Transcriptional Divergence in BMDM Cells

**Figure 2:**
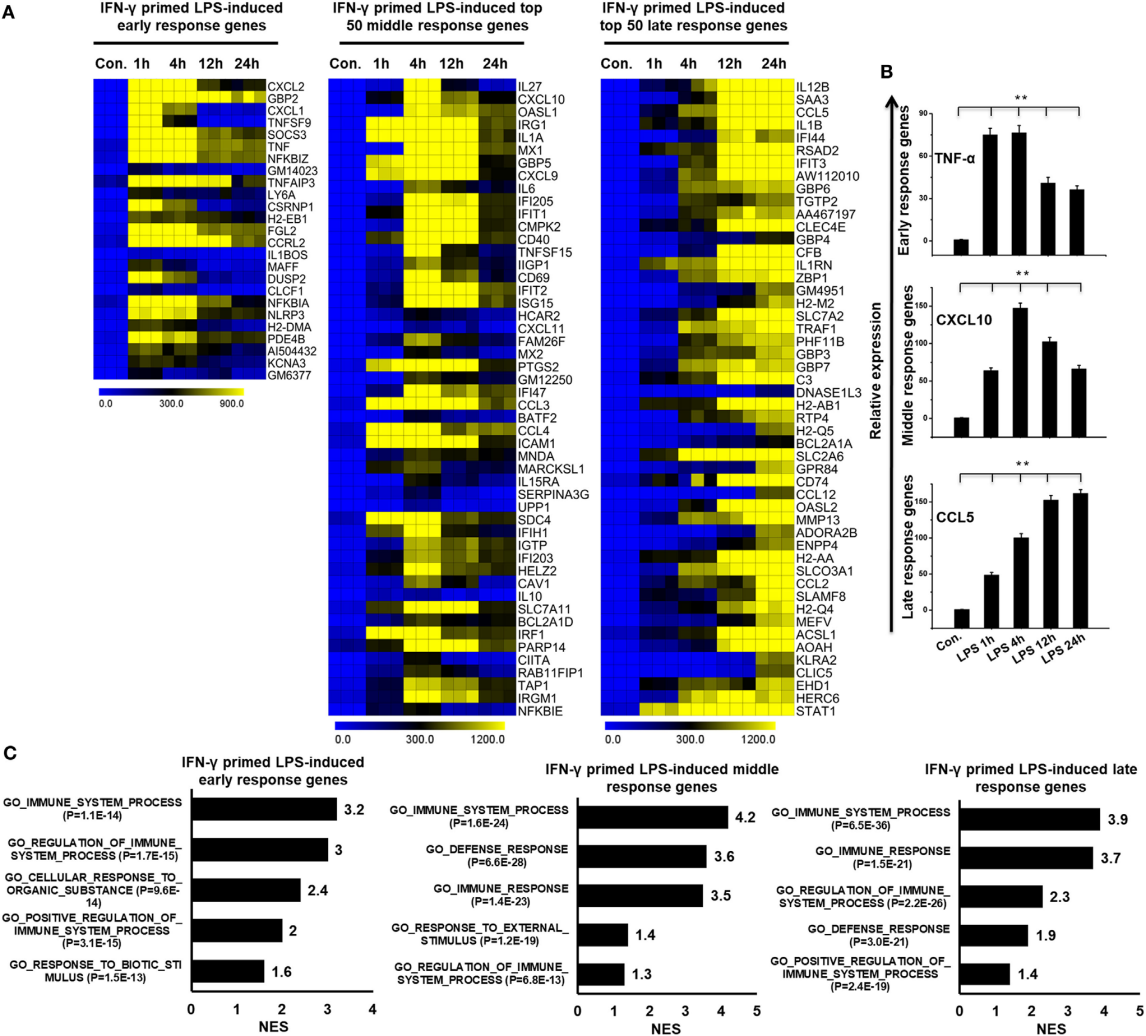
Time series of interferon-g (IFN-γ)-primed lipopolysaccharide (LPS)-induced gene expression in bone marrow-derived macrophages (BMDMs). **(A)** Heat map representation depicting the expression of early, middle, and late upregulated positive regulators of inflammatory genes in BMDMs after IFN-γ-primed LPS stimulation (*P* ≤ 0.01 and log_2_-fold change ≥ 1.5). **(B)** validation of early (TNF-α), middle (CXCL10), and late (CCL5), upregulated genes by quantitative real-time polymerase chain reaction (qRT-PCR) in BMDMs. The TNF-α, CXCL10, and CCL5 genes were significantly upregulated in IFN-γ-primed LPS-stimulated BMDMs. Gene expression was normalized to glyceraldehyde-3-phosphate dehydrogenase transcript levels. **P < 0.001 compared with the control. The data represent three independent biological experiments. **(C)** Gene set enrichment analysis (GSEA) results showing C5 gene ontology (GO) terms collections of the GSEA molecular signatures database (MSigDB) v6.1 in early, middle, and late upregulated positive regulators of inflammatory genes in BMDMs. The top GO terms are ranked by the normalized enrichment score (NES).

To globally understand IFN-γ-mediated TLR4 cross-regulation in BMDMs, the IFN-γ-primed LPS-inducible samples were evaluated using whole-genome RNA-seq (Illumina; HiSeq2000). The time course samples (IFN-γ-primed LPS 1, 4, 12, and 24 h stimulation) were subjected to whole-genome RNA-seq. Next, we performed PCA to explore relationships among the sample biological replicates. The PCA analysis revealed significant separation and a high level of coherence between the sample biological replicates in BMDMs (Figure S2A in Supplementary Material). We identified significantly altered genes over the time course after LPS stimulation. According to the above criteria, 595 genes at 1 h, 1,912 genes at 4 h, 1,526 genes at 12 h, and 1,347 genes at 24 h genes were differentially regulated in IFN-γ-primed LPS-inducible BMDMs (Figure S3A in Supplementary Material). Of these, 458, 1082, 834, and 721 genes were upregulated and 137, 830, 692, and 626 genes were downregulated at 1, 4, 12, and 12 h, respectively (Figure S3A in Supplementary Material). A group of 255 upregulated genes were shared between the four time course samples (Figure S3B in Supplementary Material). In order to obtain clusters of upregulated genes, we perform hierarchical clustering based on the shared set of transcripts (255 upregulated genes) (Figure 2A). Three well-separated gene pools were detected: “early,” upregulated at 1 h; “middle,” upregulated at 4 h; and final, “late,” upregulated at 12 and 24 h (Figures 2A,B; Figure S3C in Supplementary Material). Next, we performed functional annotation analysis for GO based on the biological processes (BP) using GSEA. The early, middle, and late upregulated genes were determined to be involved in several BPs such as immune system process, defense response, and cellular response to organic substance (Figure 2C).

**Figure 3:**
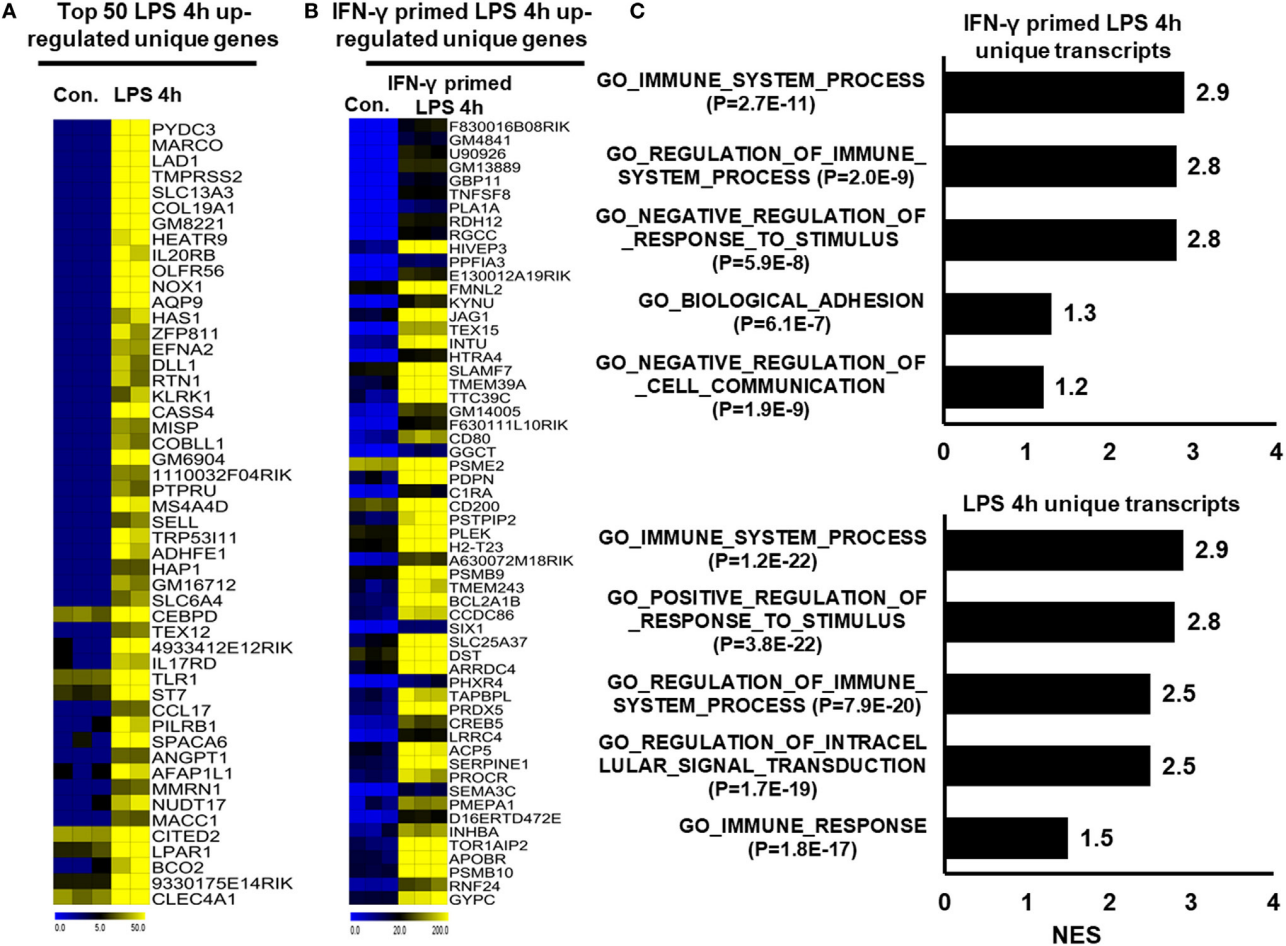

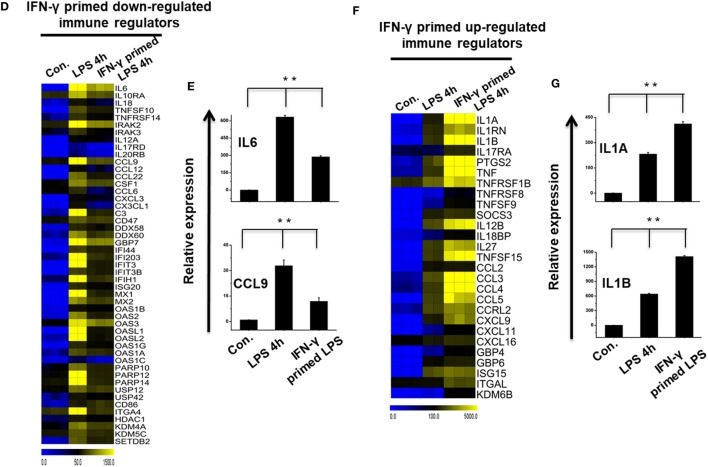
Comparison of only lipopolysaccharide (LPS)-inducible and interferon-g (IFN-γ)-primed LPS-inducible transcriptional datasets. **(A,B)** Heat map representation of the transcripts that were uniquely upregulated in LPS-inducible and IFN-γ-primed LPS-inducible bone marrow-derived macrophages (BMDMs), respectively. **(C)** Gene set enrichment analysis (GSEA) results showing C5 gene ontology (GO) terms collections of the GSEA molecular signatures database (MSigDB) v6.1 in LPS-inducible and IFN-γ-primed LPS-inducible upregulated inflammatory genes in BMDMs. The top GO terms are ranked by the NES. **(D,F)** Heat map representation of the positive regulators of inflammatory transcripts [cytokines, chemokines, and interferon regulated genes (IRGs)] that were either down or upregulated in IFN-γ-primed LPS-inducible BMDMs compared with only LPS-inducible BMDMs, respectively. **(E,G)** Validation of IFN-γ-primed LPS-inducible down or upregulated genes compared with only LPS-inducible BMDMs, respectively. Gene expression was normalized to the glyceraldehyde-3-phosphate dehydrogenase transcript levels. **P < 0.001 compared with the control. The data represent three independent biological experiments.

To further identify common and unique characteristics between IFN-γ-primed LPS-inducible and only LPS-inducible BMDMs, we again used RNA-seq dataset to compare the gene expression of IFN-γ-primed LPS-inducible with that of only LPS-inducible BMDMs. We compared the most highly expressed transcripts (>3-fold log_2_ change) at 1 and 4 h in IFN-γ-primed LPS-inducible and only LPS-inducible BMDMs. Differential expression analysis clearly revealed that IFN-γ-primed LPS-inducible, and only LPS-inducible BMDMs shared 238 transcripts at 4 h, suggesting a substantial number of similarities between the two treatment conditions (Figure S4A in Supplementary Material). IFN-γ-primed LPS-inducible and only LPS-inducible BMDMs, however, also had substantial differences in their transcriptomes. There were 58 upregulated genes in IFN-γ-primed LPS-inducible BMDMs at 4 h that were distinct from LPS-inducible BMDMs (Figure S4A in Supplementary Material). In contrast, 180 genes were upregulated in only LPS-inducible BMDMs at 4 h that were distinct from IFN-γ-primed LPS-inducible BMDMs (Figure S4A in Supplementary Material). The unique gene sets at 4 h in IFN-γ-primed LPS-inducible and only LPS-inducible BMDMs are presented in Figures 3A,B. We observed that the functions most associated with the unique genes in IFN-γ-primed LPS-inducible and only LPS-inducible BMDMs were related to the immune system process (Figure 3C). Interestingly, IFN-γ-primed LPS-inducible and only LPS-inducible upregulated unique genes were involved in biological adhesion and regulation of intracellular signal transduction, respectively (Figure 3C). The greatest differences between IFN-γ-primed LPS-inducible and only LPS-inducible upregulated genes were therefore a difference in biological adhesion and regulation of intracellular signal transduction according to GSEA.

To further characterize the similarities and differences between IFN-γ-primed LPS-inducible and Only LPS-inducible BMDMs, we compared the expression levels of several specific gene families involved in immune responses, including cytokines, chemokines, IRGs, and epigenetic regulators. We found that only LPS-inducible BMDMs expressed significantly higher levels of IL6, IL10RA, IL18, TNFSF10, CCL9, CCL12, and CCL22, among others (Figures 3D,E; Figure S4B in Supplementary Material). In contrast, IFN-γ-primed LPS-inducible BMDMs expressed significantly higher levels of several specific gene families involved in immune responses, including IL1A, IL1RN, IL1B, PTGS2, TNF-α, CCL2, CCL3, and CCL4, among others, compared with only LPS-inducible BMDMs (Figures 3F,G; Figure S4C in Supplementary Material). These data suggest that alterations in the expression levels of these transcripts during pathologic conditions not only reflect unique functional capabilities but can also be used as potential targets to identify these cells in distinct physiologic conditions.

### Time Series of the Divergence of IFN-γ-Primed LPS-Inducible and only LPS-Inducible TFs and Transcription Co-Factors (TcoFs) in BMDMs

**Figure 4:**
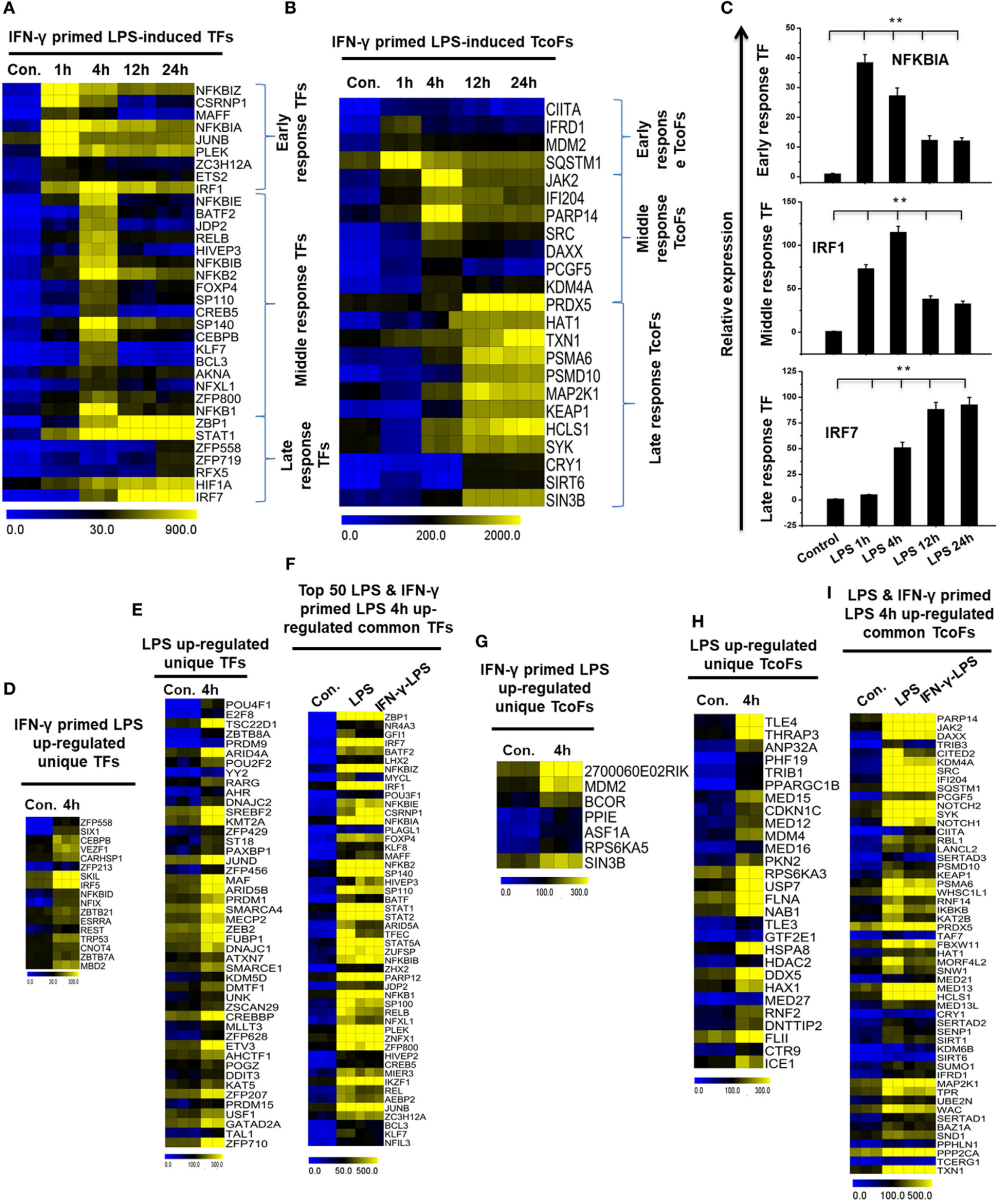
Time series and differences in transcription factor (TF) and transcription co-factor (TcoF) expression in lipopolysaccharide (LPS)-induced and interferon-g (IFN-γ)-primed LPS-induced bone marrow-derived macrophages (BMDMs). **(A,B)** Heat map representation depicting the expression of early, middle, and late upregulated TFs and TcoFs in IFN-γ-primed LPS-inducible BMDMs, respectively (*P* ≤ 0.01, and log_2_-fold change ≥ 1.0). **(C)** Validation of early (NFkBIA), middle interferon regulatory factor-1 (IRF1), and late (IRF7) upregulated TFs in IFN-γ-primed LPS-inducible BMDMs. Gene expression was normalized to glyceraldehyde-3-phosphate dehydrogenase transcript levels. **P < 0.001 compared with the control. The data represent three independent biological experiments. **(D,G)** Heat map representation of the TFs and TcoFs that were uniquely upregulated in IFN-γ-primed LPS-inducible BMDMs, respectively. **(E,H)** Heat map representation of the TFs and TcoFs that were uniquely upregulated in LPS-inducible BMDMs, respectively. **(F,I)** Heat map representation of common TFs and TcoFs in LPS-inducible and IFN-γ-primed LPS-inducible BMDMs.

Transcription factors and their targets are a key part of active gene expression programs, and the alteration of key TFs is a feature of many deadly diseases, including many inflammatory diseases (45). To identify the key TFs associated with inflammation, we computed RNA-seq dataset and analyzed the multiple families of TFs that showed a log_2_-fold change in expression ≥1.0 in IFN-γ-primed LPS-inducible BMDMs compared with control BMDMs. Based on a search of the TF database (46), we detected a total of 34 upregulated TFs that were shared between the four time course samples in Figure S5A in Supplementary Material. In order to obtain clusters of upregulated TFs, we perform hierarchical clustering based on the shared set of TFs (34 upregulated TFs). Three well-separated TFs were detected: “early,” upregulated at 1 h; “middle,” upregulated at 4 h; and final, “late,” upregulated at 12 and 24 h (Figures 4A,C; Figure S5C in Supplementary Material). Many deterministic factors, such as the localization and modification of the associated TFs, affect TF alterations. Therefore, using a similar approach (46), we next examined the TcoFs in IFN-γ-primed LPS-inducible BMDMs compared with control BMDMs. According to the above criteria, 10 TcoFs were shared between the four time course samples, and 13 TcoFs were shared between the middle and late time course in IFN-γ-primed LPS-inducible BMDMs (Figure 4B; Figure S5B in Supplementary Material).

To further identify common and unique characteristics between IFN-γ-primed LPS-inducible and only LPS-inducible BMDMs, we next examined the TFs and TcoFs in IFN-γ-primed LPS-inducible compared with only LPS-inducible BMDMs. Using a similar approach (46), we found that IFN-γ-primed LPS-inducible and only LPS-inducible BMDMs shared 120 TFs and 53 TcoFs (log_2_-fold change ≥ 1.0, *P* ≤ 0.01) at 4 h time, respectively, suggesting a substantial number of similarities between the two BMDM treatment conditions (Figures 4F,I; Figures S5D,E in Supplementary Material). However, IFN-γ-primed LPS-inducible and only LPS-inducible BMDMs also displayed substantial differences in transcriptional regulators. In IFN-γ-primed LPS-inducible BMDMs, 17 TFs and 7 TcoFs were upregulated that were not common to the only LPS-inducible BMDMs. In contrast, in only LPS-inducible BMDMs, 46 TFs and 28 TcoFs were upregulated at 4 h that were not common to the IFN-γ-primed LPS-inducible BMDMs (Figures 4D,E,G,H; Figures S5D,E in Supplementary Material). The unique and common sets of TFs and TcoFs at 4 h are presented in Figures 4D–I. These data suggest that the identified TFs and TcoFs have an important role of the selective inflammatory gene expression that occurs in IFN-γ-primed LPS-inducible and only LPS-inducible BMDMs.

### Identification of Important Motif Activities Involved in IFN-γ-Primed LPS-Inducible BMDMs

**Figure 5:**
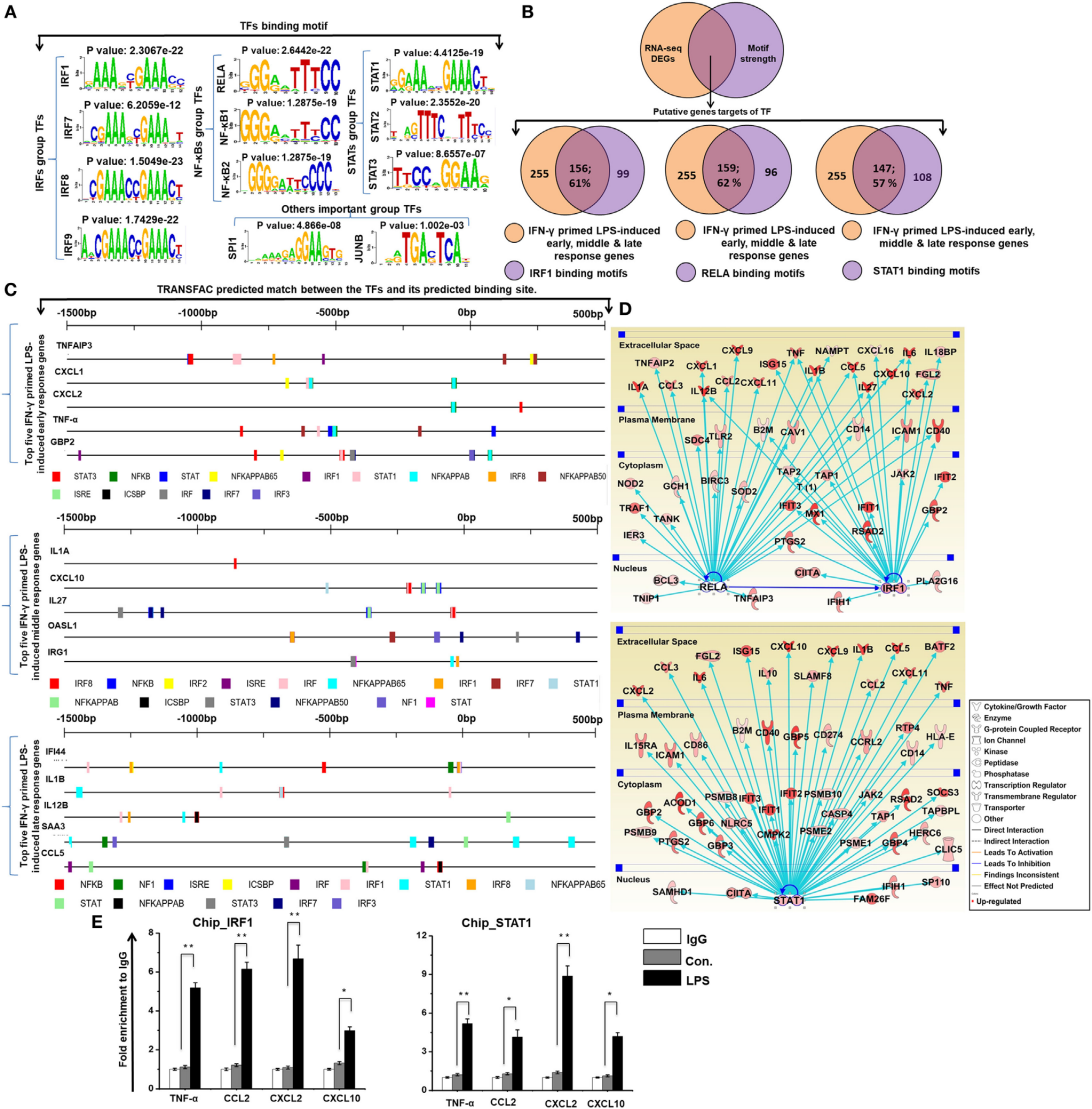
Transcription factor (TFBSs) analysis within the promoters of common early, middle, and late upregulated genes in interferon-g (IFN-γ)-primed lipopolysaccharide (LPS)-inducible bone marrow-derived macrophages (BMDMs). **(A)** Patterns of TF motif enrichment within the promoters of the common early, middle and late upregulated genes in IFN-γ-primed LPS-inducible BMDMs **(B)** Venn diagrams of common early, middle and late upregulated genes associated with interferon regulatory factor-1 (IRF1), RELA, and signal transducer and activator of transcription 1 (STAT1) in BMDMs. **(C)** TRANSFAC predicted match showing the predicted TFs and their predicted binding sites for the top five early, middle and late upregulated genes in IFN-γ-primed LPS-inducible BMDMs. **(D)** The activity of highly connected positive regulators of the inflammatory genes IRF1, RELA, and STAT1 led to the activation of this network, as assessed using the ingenuity pathway analysis molecule activity predictor in IFN-γ-primed LPS-inducible BMDMs. **(E)** Chromatin Immunoprecipitation (ChIP) assay to determine the binding of IRF1, and STAT1 to target genes. The ChIP-enriched samples were subjected to quantitative PCR with selected genes promoters. The graphs represent the mean fold values of enrichment relative to IgG control from three independent experiments. *P ≤ 0.01 and **P ≤ 0.001 compared with the control.

To investigate the contribution of the promoter sequence and the underlying mechanisms of the shared set of transcripts (255 upregulated genes) among the four time course samples of BMDMs (IFN-γ-primed LPS 1, 4, 12, and 24 h stimulation), we precisely evaluated DNA-binding factors within the promoter sequences of the coordinately expressed genes. We used the Pscan software tool of reported TF binding sites in parallel with expression profiling, which was applied to perform computational promoter analysis of over-represented *cis*-motifs residing within the 5′-promoter regions of IFN-γ-primed LPS-inducible upregulated genes. We identified the putative binding sites for IRF group TFs (IRF1, IRF7, IRF8, and IRF9), NF-κB group TFs (RELA, NF-κB1, and NF-κB2), and STAT group TFs (STAT1, STAT2, and STAT2), as well as other important groups of TFs (SPI1 and JUNB) that were significantly enriched in the IFN-γ-primed LPS-inducible BMDMs (Figure 5A). Next, we analyzed the number of IFN-γ-primed LPS-inducible shared sets of upregulated genes containing IRF1, RELA, and STAT1-binding motifs in their promoter sequences. Interestingly, among the upregulated genes, we found that a significant percentage of the cytokines and chemokines, as well as IRGs, contained IRF1 (156/255; 61%), RELA (159/255; 62%), and STAT1 (147/255; 57%) binding motifs within the promoter region of upregulated genes (from −950 bp to +50 bp), as indicated in Figure 5B and Excel file S1 in Supplementary Material. In addition to the Pscan software tool, we also designed a method for discovering the motif conservation in mammalian promoters using the TRANSFAC database with mapped annotated binding sites (TFBSs), which were experimentally defined promoters from transcription start site (TSS) region. We conducted a TFBS analysis to assess the top five early, middle, and late upregulated genes in IFN-γ-primed LPS-inducible BMDMs. Applying this TRANSFAC database to the gene promoters revealed that the putative binding sites for IRF, NF-κB, and STAT were significantly enriched (Figure 5C). Furthermore, in order to obtain target genes that were directly or indirectly activated by the identified TFs, we applied IPA software. Importantly, the assessment of upstream regulators by IPA, similarly revealed that most of the cytokines and chemokines were also directly regulated by TFs, including RELA, IRF1, and STAT1, among the shared set of upregulated genes (Figure 5D). In addition, we performed ChIP experiments in order to determine IRF1 and STAT1 occupancy in target genes promoter regions. IRF1 and STAT1 directly bound to TNF-α, CCL2, CXCL2, and CXCL10 promoter regions in the LPS-induced RAW264.7 macrophage cell line (Figure 5E). In total, these data indicate that inducible expressed, distinct or overlapping sets of TF family proteins, as well as regulatory promoter sequences, may affect the transcriptional activity of a gene under distinct pathologic conditions in BMDMs.

### Transcriptional Divergence in IL-4 and IL-13-Treated BMDMs

**Figure 6:**
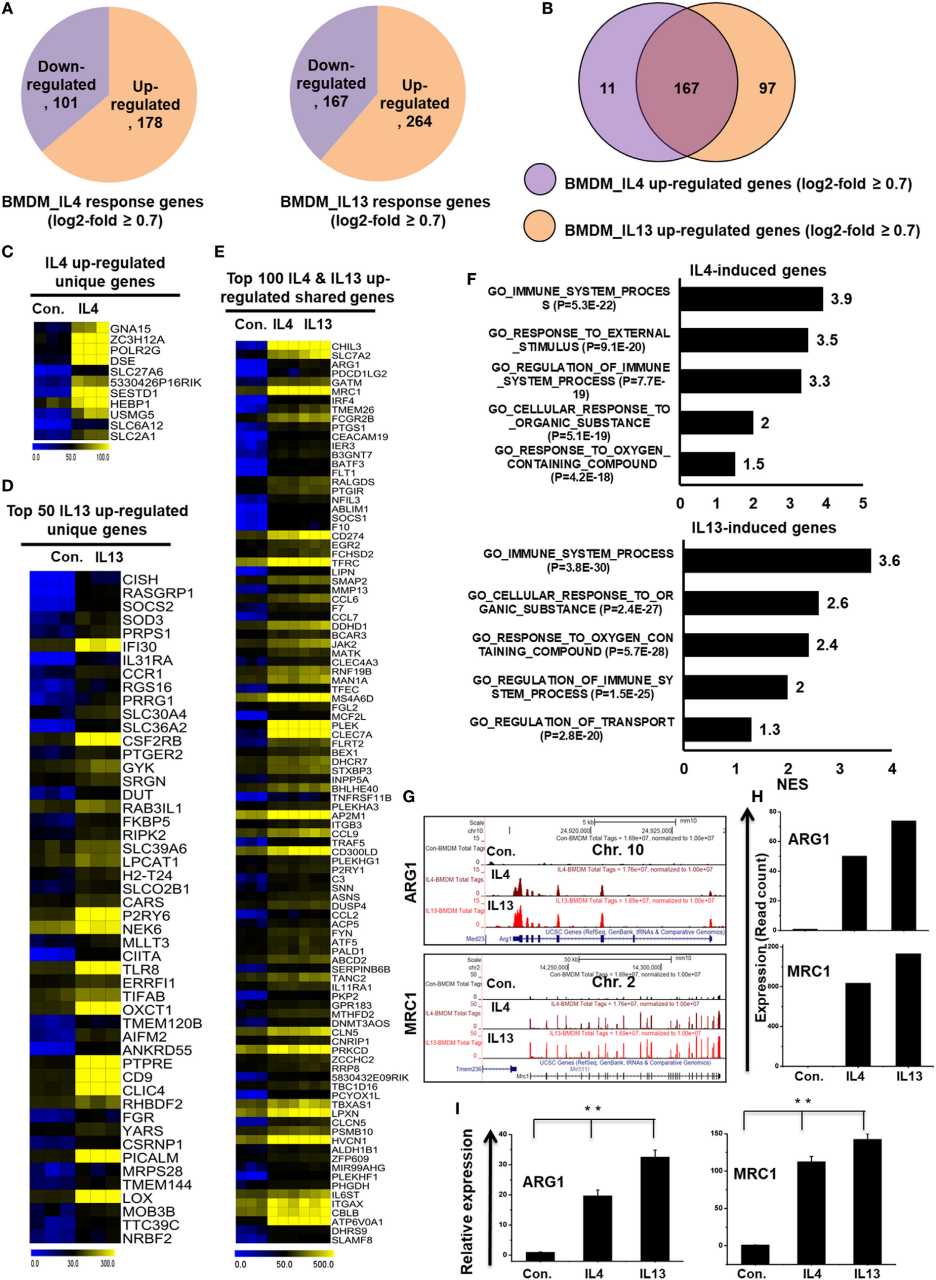
Differences in transcriptomic profiles between interleukin-4 (IL)-4 and IL-13-treated bone marrow-derived macrophages (BMDMs). **(A)** Pie chart displaying the number of up or downregulated genes in IL-4 and IL-13 treated BMDMs. **(B)** The area of overlap indicates the number of unique or shared upregulated genes in IL-4 and IL-13-treated BMDMs. **(C,D)** Heat map representation of the transcripts that were uniquely upregulated in IL-4 and IL-13-treated BMDMs, respectively. **(E)** Heat map representation depicting transcripts that were commonly expressed in IL-4 and IL-13-treated BMDMs. **(F)** Gene set enrichment analysis results of the functional annotations that were associated with the upregulated genes in IL-4 and IL-13-treated BMDMs. **(G)** UCSC Browser images representing the normalized RNA-seq read density in commonly expressed M2-associated genes between IL-4 and IL-13-treated BMDMs. **(H)** The transcript abundance (in read count) was evaluated using RNA-seq for commonly expressed M2-associated genes between IL-4 and IL-13-treated BMDMs. **(I)** Validation of commonly upregulated M2-associated genes in IL-4 and IL-13-treated BMDMs. Gene expression was normalized to glyceraldehyde-3-phosphate dehydrogenase transcript levels. **P < 0.001 compared with the control. The data represent three independent biological experiments.

To directly compare the transcriptomes of IL-4-treated and IL-13-treated BMDMs, RNA-seq experiments were carried out after depletion of rRNA. Next, to investigate the congruency among biological replicates and general trends in the data, PCA was performed. The PCA analysis showed no outlier samples and separate grouping of control BMDMs and IL-4 and IL-13-stimulated BMDMs (Figure S2B in Supplementary Material). Using a false discovery rate (FDR ≤ 0.01), *P* ≤ 0.01, and fold change ≥0.7 log_2_ as cutoff values for the up or downregulation of genes, we identified 279 genes that were differentially regulated in IL-4-treated BMDMs (Figure 6A). Among 279 DEGs in IL-4-treated BMDMs, 178 genes were upregulated and 101 genes were downregulated (Figure 6A). Surprisingly, we found significant generational differences in IL-13-treated BMDMs, in which 431 genes were differentially regulated. Of these, 264 genes were upregulated and 167 genes were downregulated in response to IL-13 (Figure 6A). Notably, in both groups, the number of upregulated genes was approximately 1.5 times higher than that of downregulated genes. Moreover, the numbers of total differentially expressed, upregulated, and downregulated genes were comparable between the two treatments. To gain insight into the physiological function of M2 macrophages, based on our identified IL-4 and IL-13-treated molecular signature in BMDMs, we applied GSEA and IPA. We observed that the functions most associated with IL-13-treated BMDMs were involved in several BPs such as immune system process, response to external stimulus, and cellular response to organic substance (Figure 6F).

To further investigate common and unique characteristics between IL-4 and IL-13-treated BMDMs, Venn diagrams were used to compare the transcriptomes of IL-4-treated and IL-13-treated BMDMs. Using a similar approach (see above section), the differential expression analysis clearly revealed that IL-4 and IL-13 upregulated unique gene expression patterns in BMDMs (Figure 6B), indicating a substantial number of differences in their transcriptomes. In IL-13-treated BMDMs, 97 genes were upregulated that were not common to the IL-4-treated BMDMs. In contrast, in IL-4-treated BMDMs, 11 genes were upregulated that were not common to the IL-13-treated BMDMs (Figure 6B). The unique gene sets for IL-4 and IL-13-treated BMDMs are presented in Figures 6C,D. However, IL-4 and IL-13-treated BMDMs also displayed similarities in their transcriptomes. Among the upregulated genes, two treatment groups shared 167 genes (Figures 6E,G–I). We also found that almost all the M2-associated genes were more highly induced in IL-13-treated BMDMs than in IL-4-treated BMDMs (Figures 6G–I). These data suggest that IL-13 treatment induces the expression of a unique set of genes in BMDCs that are distinct from those induced by IL-4, potentially offering targets for further investigations into the biology of alternative activated macrophages.

### Paradox of Promoter Conservation and Divergence of the Expression of TFs with TcoFs in IL-4 and IL-13-Treated BMDMs

**Figure 7:**
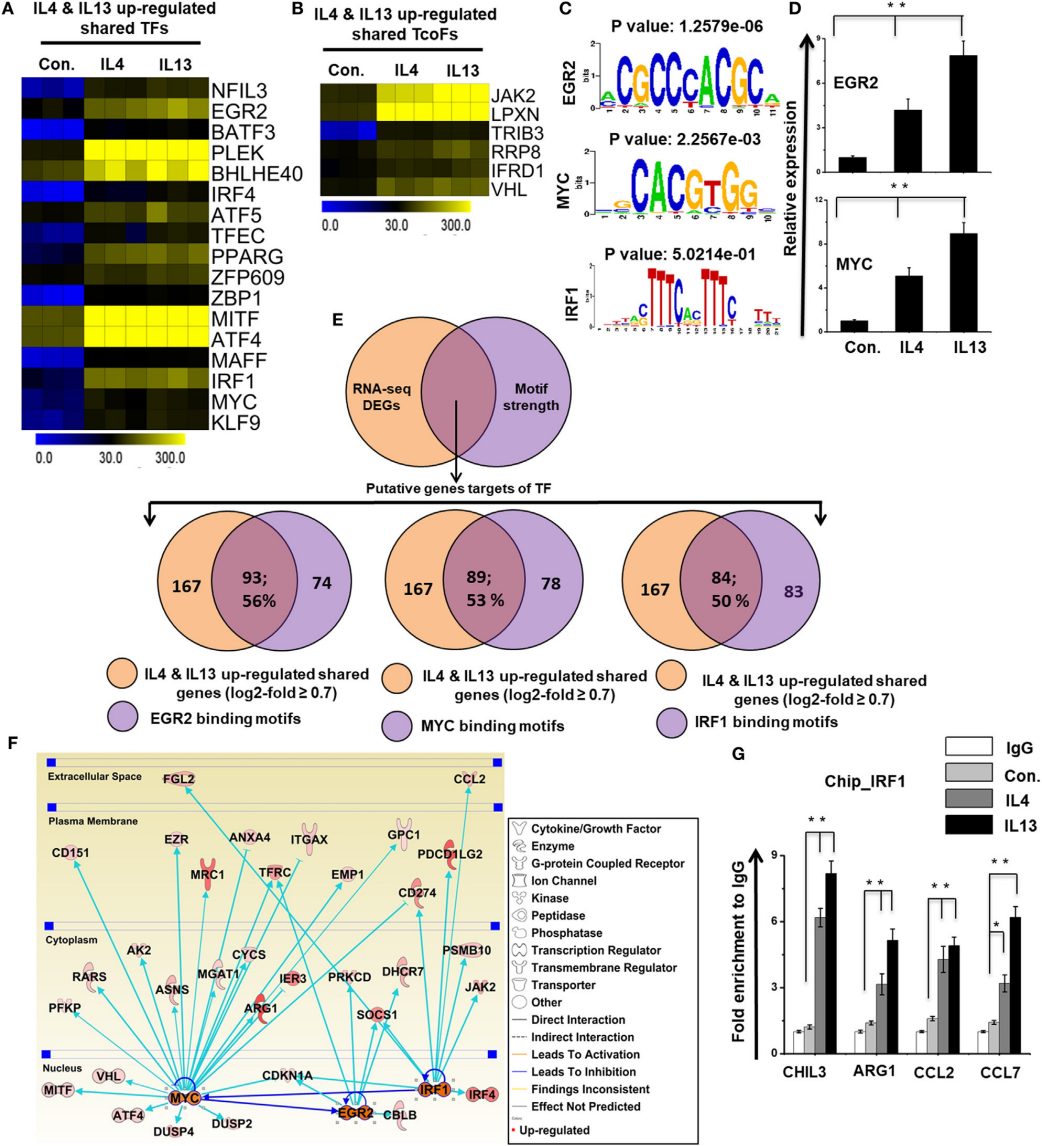
Differences in the expression of selected transcription factors (TFs) and transcription co-factors (TcoFs) between interleukin (IL)-4 and IL-13-treated bone marrow-derived macrophages (BMDMs). **(A,B)** Heat map representation of TFs and TcoFs that were commonly upregulated in IL-4 and IL-13-treated BMDMs, respectively. **(C)** Patterns of TF motif enrichment within the promoters of the common upregulated genes in IL-4 and IL-13-treated BMDMs. **(D)** Validation of commonly upregulated TFs in IL-4 and IL-13-treated BMDMs. Gene expression was normalized to glyceraldehyde-3-phosphate dehydrogenase transcript levels. **P < 0.001 compared with the control. The data represent three independent biological experiments. **(E)** Venn diagrams of common upregulated genes associated with EGR2, MYC, and IRF1 in IL-4 and IL-13-treated BMDMs. **(F)** The activity of the highly associated M2-associated genes EGR2, MYC, and IRF1 led to the activation of this network, as assessed using the ingenuity pathway analysis molecule activity predictor in IL-4 and IL-13-treated BMDMs. **(G)** Chromatin Immunoprecipitation (ChIP) assay to determine the binding of IRF1 to target genes. The ChIP-enriched samples were subjected to quantitative PCR with selected genes promoters. The graphs represent the mean fold values of enrichment relative to IgG control from three independent experiments. *P ≤ 0.01 and **P ≤ 0.001 compared with the control.

To identify the distinct sets of TFs and TcoFs in M2 responsible for the transcriptional machinery, we computed RNA-seq dataset and analyzed the multiple families of TFs and TcoFs that were expressed 0.7-log2-fold higher in IL-4 and IL-13-treated BMDMs than in control BMDMs. Based on a search of the TF database (46), we identified TFs (*n* = 18 and 22, respectively) and TcoFs (*n* = 6 and 9, respectively) that were upregulated relative to the controls (Figures S6A,B in Supplementary Material). Differential expression analysis of TFs and TcoFs clearly revealed that IL-4 and IL-13-treated BMDMs shared 17 TFs and 6 TcoFs, suggesting substantial similarities in their transcriptional regulation (Figures S6A,B in Supplementary Material). However, IL-4 and IL-13-treated BMDMs also displayed differences between the two BMDM treatment conditions. In IL-13-treated BMDMs, 5 TFs and 3 TcoFs were upregulated that were not common to the IL-4-treated BMDMs (Figures S6A,B in Supplementary Material). In contrast, in IL-4-treated BMDM cells, 1 TF was upregulated that was not common to the IL-13-treated BMDMs (Figure S6A in Supplementary Material). The shared sets of TFs and TcoFs in IL-4 and IL-13-treated BMDMs are presented in Figures 7A,B,D and Figure S6C in Supplementary Material. We also found that most important M2-associated TFs were more highly induced in IL-13-treated BMDMs than in IL-4-treated BMDMs (Figures 7A,B,D; Figure S6C in Supplementary Material).

To identify the computational promoter analysis of over-represented *cis*-motifs and the underlying mechanisms between the two treatment conditions, we precisely extracted DNA-binding factors within the corresponding promoter sequences of the coordinately expressed M2 macrophage-associated genes (167 shared genes) in IL-4 and IL-13-treated BMDMs. We found that the putative binding sites for EGR2, MYC and IRF1 were significantly enriched in the shared M2 macrophage-associated genes (Figure 7C). Next, we determined the number of shared M2 macrophage-associated genes containing EGR2, MYC and IRF1 binding motifs in the promoter sequence. Interestingly, among the upregulated genes, a significant percentage of the M2 macrophage-associated genes had EGR2 (93/167; 56%), MYC (89/167; 53%), and IRF1 (84/167; 50%) binding motifs within the promoter region (from −950 bp to +50 bp), as summarized in Figure 7E and Excel file S2 in Supplementary Material. Furthermore, in order to obtain target genes that were directly or indirectly activated by the identified TFs in response to IL-4 and IL-13 treatment, we applied IPA software. Importantly, the assessment of upstream regulators by IPA similarly revealed that the expression levels of most M2 macrophage-associated genes were also directly regulated by TFs, including EGR2, MYC and IRF1 in IL-4 and IL-13-treated BMDMs (Figure 7F). In addition, we performed ChIP experiments in order to determine IRF1 occupancy in target genes promoter regions. IRF1 directly bound to CHIL3, ARG1, CCL2, and CCL7 promoter regions in the IL-4 and IL-13-induced RAW264.7 macrophage cell line (Figure 7G). Taken together, these data may indicate that an inducible expression of distinct or overlapping sets of TF family proteins, as well as regulatory promoter sequences, may affect the capacity of a gene to alter its transcriptional level under the distinct transcriptional regulatory networks of M2 (IL-4) and M2 (IL-13).

### Macrophage Cell Lines Do Not Express a BMDM Signature

**Figure 8:**
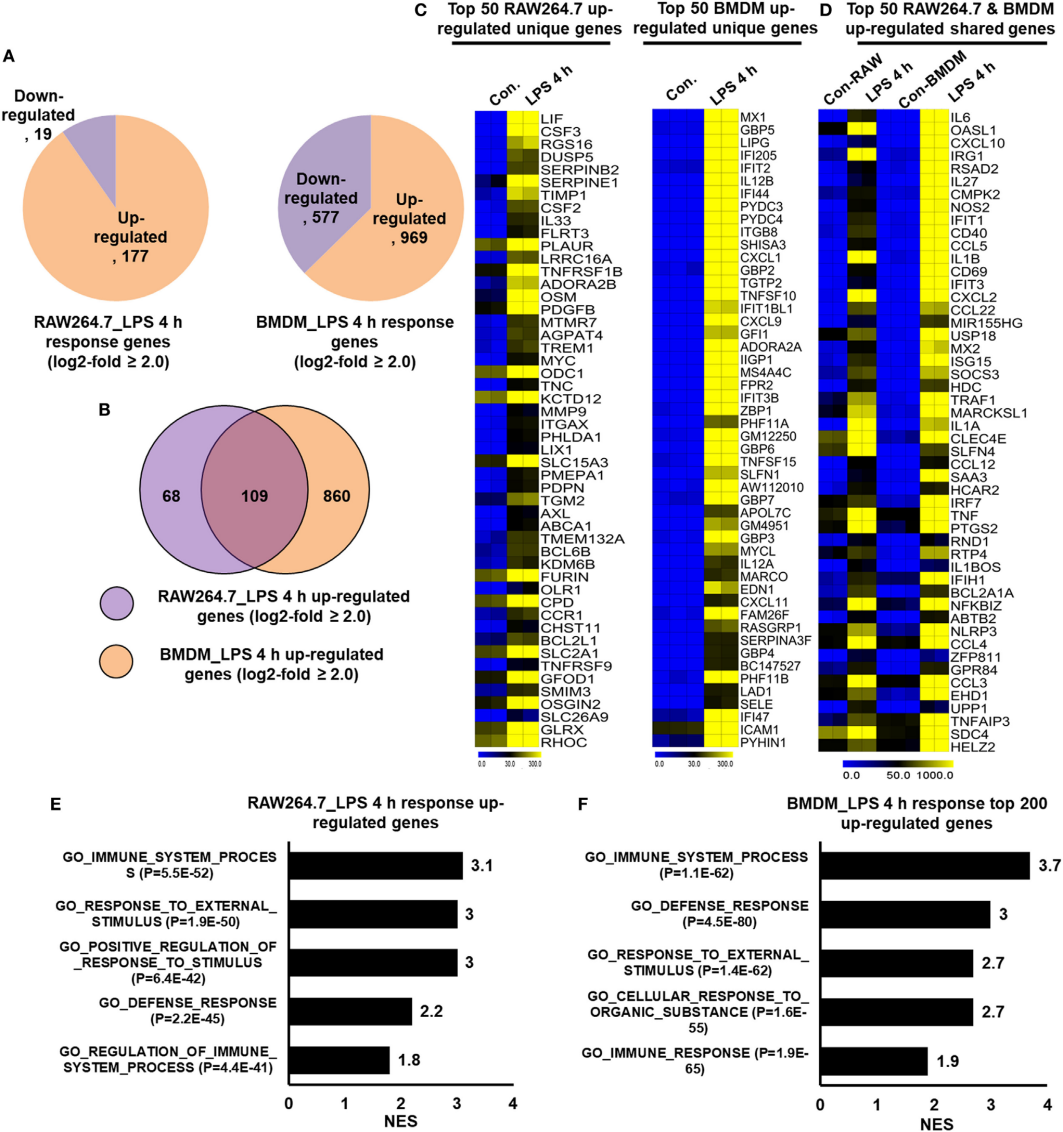
RNA-seq analyses reveals lipopolysaccharide (LPS)-induced inflammatory response-related genes and their downstream effectors in RAW264.7 macrophages and bone marrow-derived macrophages (BMDMs). **(A)** Pie chart displaying the number of up or downregulated genes at 4 h after LPS stimulation in RAW264.7 macrophages and BMDMs. **(B)** The area of overlap indicates the number of unique or shared upregulated genes after 4 h of LPS stimulation in RAW264.7 macrophages and BMDMs. **(C)** Heat map representation of the transcripts that were uniquely upregulated in RAW264.7 macrophages and BMDMs. **(D)** Heat map representation of the transcripts that were commonly upregulated in RAW264.7 macrophages and BMDMs. **(E,F)** Gene set enrichment analysis results of the functional annotations that were associated with upregulated genes at 4 h after LPS stimulation in RAW264.7 macrophages and BMDMs.

In many studies, researchers utilized the RAW264.7 macrophage cell line to understanding the complex role of macrophages in inflammation. However, doubt has arisen referring to the role of RAW264.7 macrophage cell line as a model for inflammation study. To identify the degree to which the primary BMDM genes that we detected were upregulated in RAW264.7 macrophages, we further constructed RNA-seq dataset to compare the differentially regulated genes in RAW264.7 macrophage cell line with that of primary BMDMs. Using Venn diagrams, heat maps and PCA of RAW264.7 macrophage and BMDM transcriptome profiles, we found that RAW264.7 macrophages differed from BMDMs (Figure S2C in Supplementary Material). We compared the identified molecular signatures of the most highly specific macrophage genes (log_2_-fold change ≥ 2.0) that we found to be expressed in BMDMs to those of RAW264.7 macrophages. Using a false discovery rate (FDR ≤ 0.01), *P* ≤ 0.01, and log_2_-fold change ≥2.0 as cutoff values for the up or downregulation of genes, we identified 196 genes that were differentially regulated following LPS treatment in RAW264.7 macrophages (Figure 8A). Of 196 DEGs in RAW264.7 macrophages, 177 genes were upregulated and 19 genes were downregulated (Figure 8A). Surprisingly, significant generational differences were observed at 4 h in LPS-treated BMDMs, in which 1,546 genes were differentially regulated. Of these, 969 genes were upregulated and 577 genes were downregulated following LPS treatment (Figure 8A). Our results clearly revealed that LPS upregulated unique gene expression patterns in BMDMs and RAW264.7 macrophages (Figure 8B), indicating a substantial number of differences in their transcriptomes between the two cell types. In BMDMs, 860 genes were upregulated that were not common to RAW264.7 macrophages. In contrast, 68 genes were upregulated in RAW264.7 macrophages that were not common to BMDMs (Figure 8B). The unique gene sets are presented in Figure 8C. However, BMDMs and RAW264.7 macrophages also had similarities in their transcriptomes and physiological functions according to GSEA (Figures 8D–F). Among the upregulated genes, RAW264.7 macrophages and BMDMs shared 109 genes following LPS treatment (Figure 8D).

**Figure 9:**
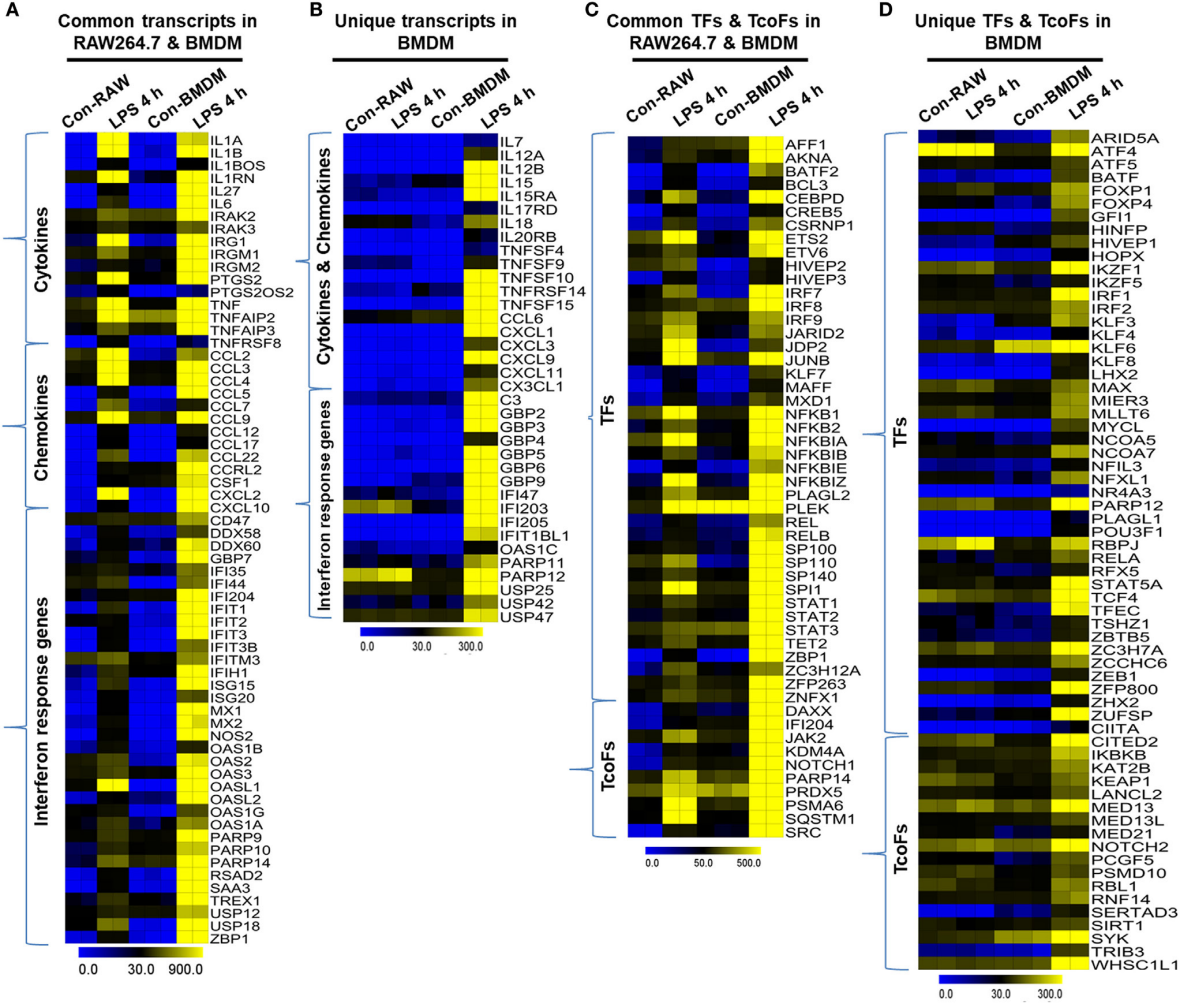

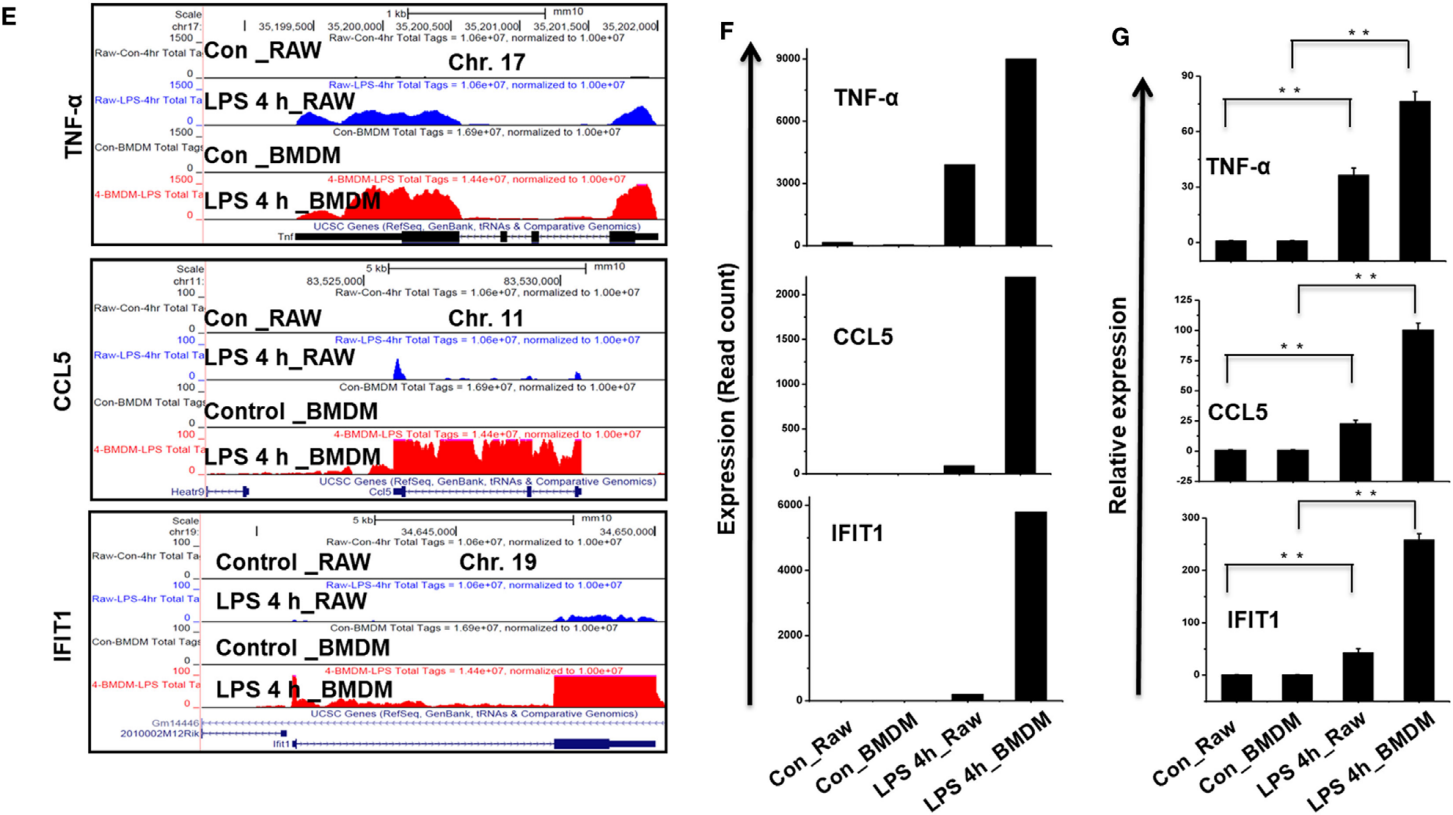
Differences in the transcriptomic profiles [cytokines, chemokines, interferon regulated genes (IRGs), transcription factors (TFs) and transcription co-factors (TcoFs)] between established RAW264.7 macrophages and bone marrow-derived macrophages (BMDMs). **(A,C)** Heat map representation depicting the common expression of positive regulators of inflammatory genes and TFs/TcoFs between RAW264.7 macrophages and BMDMs at 4 h after lipopolysaccharide (LPS) stimulation, respectively. **(B,D)** Heat map representation depicting the unique expression of positive regulators of inflammatory genes and TFs/TcoFs between RAW264.7 macrophages and BMDMs at 4 h after LPS stimulation, respectively. **(E)** UCSC Browser images representing the normalized RNA-seq read density for commonly expressed positive regulators of inflammatory genes between RAW264.7 macrophages and BMDMs. **(F)** The transcript abundance (in read count) was evaluated using RNA-seq to identify commonly expressed positive regulators of inflammatory genes between RAW264.7 macrophages and BMDMs. **(G)** quantitative real-time polymerase chain reaction (qRT-PCR) analysis of LPS-induced positive regulators of inflammatory gene expression (cytokines, chemokines, and interferon response genes) that were common to RAW264.7 macrophages and BMDMs. Gene expression was normalized to glyceraldehyde-3-phosphate dehydrogenase transcript levels. **P < 0.001 compared to the control. The data represent three biologically independent experiments.

Importantly, we identified the expression of several specific inflammation-related gene families, as well as TFs and TcoFs, which were uniquely upregulated in LPS-treated BMDMs. We found that LPS upregulated 10 unique cytokines, 9 chemokines, 13 IRGs, 47 TFs, and 18 TcoFs in BMDMs (Figures 9B,D). The following inflammation-related genes were upregulated only in BMDMs: cytokines/chemokines (IL7, IL12A, IL12B, IL15, CCL6, CXCL1, CXCL3, CXCL9, etc.), IRGs (C3, GBP2, GBP3, GBP4, GBP5, GBP6, GBP9, GBP10, among others), TFs (ATF4, ATF5, BATF, IRF1, IRF2, among others), and TcoFs (MED13, MED13L, SIRT1, among others). However, BMDMs and RAW264.7 macrophages also elicited the induction of shared cytokines, chemokines, IRGs, TFs, and TcoFs (Figures 9A,C). We also found that most of the positive regulators of inflammatory genes were more highly induced in BMDMs than in RAW264.7 macrophages (Figures 9E–G). Thus, BMDMs and RAW264.7 macrophages maintain their own molecular signature during inflammatory responses.

### Confirmation of DEGs by qRT-PCR

Next, we confirmed the expression of the DEGs by real-time qRT-PCR using GAPDH as a reference gene. Most of the genes were selected for validation based on the distinct effects of IFN-γ-primed LPS-inducible versus only LPS-inducible BMDMs; IL-4 and IL-13-treated BMDMs as well as LPS-inducible BMDMs versus RAW264.7 macrophages. In almost all cases qRT-PCR results were consistent with the RNA-seq dataset based on the direction of change as well as its magnitude (Figures 2B, 3E,G, 4C, 6I, 7D and 9G).

## Discussion

In the present study, using high-resolution transcriptome analysis, we showed that LPS and IFN-γ reprogrammed the macrophage transcriptome to alter inflammatory responses. Although other laboratories have reported to determine gene expression changes in macrophages (21–24), thus far, a genome-wide search for IFN-γ mediated TLR4 cross-regulation has not been performed in murine macrophages. The aim of the present study was to provide more comprehensive transcriptome profiling of macrophages to inflammatory stimulation, was enhanced through the use of RNA-seq technology to examine IFN-γ-mediated TLR4 cross-regulation, IL-4/IL-13-primed M2 macrophage polarization and differences between RAW264.7 macrophages and BMDMs. The data obtained herein to a larger extent compared with the other literature studies (21–24). The current study not only significantly extends previous research findings but also distinguishes between IL-4 and IL-13-primed M2 macrophage polarization and between RAW264.7 macrophages and BMDMs. Such data support the contention that signaling crosstalk between IFN-γ and LPS to cross-regulate transcriptional responses, qualitative difference between RAW264.7 macrophages and BMDMs as well as IL-4 and IL-13-primed M2 macrophage polarization are prominent components of innate immunity that may lead to inflammation.

In the analysis of IFN-γ-primed LPS-induced genes, we identified three sets of genes: early, middle and late response genes. The most highly enriched groups of genes in the early response group are known to be involved in the immune system process and cellular response to organic substance: for example, CXCL2, GBP2, CXCL1, TNFSF9, SOCS3, and TNFA, among others. The genes in the middle response group are known to be involved in the immune system process and defense response: for example, IL27, CXCL10, OASL1, IRG1, IL1A, and MX1, among others. Late response genes are known to be involved in immune system process: for example, IL12B, SAA3, CCL5, IL1B, IFI44, and RASD2 (Figures 2A,C). In our study, we also found that a set of TFs, including NF-κB, STAT, KLF, BATF, ETS, and FOXP, likely plays an important in macrophage activation. Importantly, we identified several early induced TFs, including NF-κBIA, and JUNB; middle induced TFs, including IRF1, and BATF2; and late induced TFs, including STAT1, and IRF7 (Figure 4A), indicating that these TFs might controls time-dependent regulation of inflammatory response genes. Additionally, our RNA-seq technology identified that several TcoFs were highly expressed in BMDMs, which are critical mediators of inflammatory diseases (Figure 4B).

In many studies, researchers used IFN-γ for activating macrophages that is intimately engaged innate immune response (7, 47, 48). IFN-γ-induced priming of TLR responses can escalate macrophage proinflammatory mediator’s secretion (8, 9, 48). In this respect, IFN-γ are promising innate immune targets, since they are known to modulate proinflammatory mediator’s secretion that affect innate immunity and may lead to inflammation. IFN-γ engagement of TLRs leads to pleiotropic immunostimulatory and immunosuppressive effects (10, 11). However, the dynamic outcome of genome-wide approaches and the molecular mechanisms underlying this synergy in murine macrophages remain largely unaddressed and are under active investigation. Our genome-wide analysis using the major experimental uses of macrophages, along with the integration with multiple gene sets and bioinformatics analysis, provides the most robust and comprehensive assessment to date of IFN-γ-mediated TLR4 cross-regulation at the level of the macrophage transcriptome. The data obtained herein to a larger extent compared with the other literature studies (10, 12). The current study not only significantly extends previous research findings but also identifies a distinct mechanism whereby IFN-γ either augments or suppresses the expression of immune-inflammatory genes in BMDMs. In our study, we found that a set of unique TFs as well as TcoFs were largely affected either by IFN-γ-primed LPS stimulation or only LPS stimulation in macrophages cells, suggesting that these unique TFs and TcoFs might controls regulation of IFN-γ-primed immunostimulatory and immunosuppressive effects (Figures S5D,E in Supplementary Material). Nevertheless, whether these TFs and TcoFs have any immunostimulatory or immunosuppressive roles in IFN-γ-primed modulation of macrophage activation will require experimental validation using knockouts or overexpression models.

Few empirical studies have compared the effects of IL-4 and IL-13 *in vitro* and *in vivo* (49, 50). However, thus far a genome-wide search and the similarities and differences between the effects to those stimuli on BMDMs gene expression have not been fully characterized. Strikingly, BMDMs showed enhanced reactivity to IL-13 and, therefore, number of transcript alterations was greater, including many unidentified transcripts, compared with IL-4-induced BMDMs. Therefore, there are emerging differences between IL-4 and IL-13-induced BMDMs. Both the large number of DEGs and fold changes of commonly altered M2-associated genes showed significantly greater modulation in IL-13-induced BMDMs compared with IL-4-induced BMDMs. In IL-13-induced BMDMs, 97 genes were upregulated that were not common to the IL-4-induced BMDMs (Figure 6B). Importantly, the RNA-seq approach taken in the present study was first step to identify, the differentially expressed TFs and TcoFs that were not identified in response to IL-4 activation, but that were found to be altered in IL-13-induced BMDMs. The following were markedly affected only in IL-13-induced BMDMs: TFs (MLLT3, CSRNP1, MYRF, AHR, and KLF10); TcoFs (CIITA, GNL3, and PICALM). In addition, the extent of the fold changes of commonly altered M2-associated TFs (EGR2, MYC, IRF4, among others) showed significantly greater modulation in IL-13-induced BMDMs compared with IL-4-induced BMDMs. Our data are consistent with previous research findings showing that EGR2 and MYC are critical M2 macrophage markers (26). In our RNA-seq analysis, we also identified several other TFs (NFIL3, BATF3, IRF4, ATF5, among others) in IL-4 and IL-13-induced BMDMs. Each of these TFs (EGR2, MYC, IRF1, among others) is predicted to control central aspects of the IL-4 and IL-13 response and may represent candidates for experimental validation using knockout or overexpression models.

The RAW264.7 macrophage cell line was originally derived from Abelson murine leukemia virus-infected Balb/c mice (51), and we have witnessed an explosion of work in the macrophage field over the past decade based on a PubMed search. In recent years, RAW264.7 macrophages have been the most frequently used cell type in proteomic analyses (52), cytokine response analyses (53), and the identification of DNA receptors (54). However, doubt has arisen regarding the importance of this cell line, about the use of model systems. There are important differences between RAW264.7 macrophages and BMDMs. For example, transfection with Abelson murine leukemia virus renders these cells, different in some ways from primary macrophages (55). Recently, other laboratories have reported proteomic analyses of RAW264.7 macrophages that revealed many dis-similarities compared with primary BMDMs (56). Using this array, we identified cytokines/chemokines, antiviral genes, and IRGs that were significantly enriched and associated with the inflammatory response, and these alterations were greater in BMDMs compared with RAW264.7 macrophages (Figures 8C,D). Importantly, the RNA-seq approach taken in this study was first step to identify several important differentially expressed cytokines/chemokines, antiviral genes, and IRGs that were not previously detected to be activated by LPS in RAW264.7 macrophages, but that were found to be altered in BMDMs (Figure 9B). We also identified increased expression of important TFs associated with the immune response, and we found that these alterations were greater in BMDMs compared with RAW264.7 macrophages (Figure 9D). More importantly, we identified several TFs, including ATF4, ATF5, BATF, FOXP1, FOXP4, IRF1, and IRF2, among others, whose expression profiles were altered uniquely in BMDMs (Figure 9D), indicating that these TFs might control transcriptional regulation of inflammatory responses. In addition, our RNA-seq analysis revealed that these and other TFs were expressed to a greater degree in BMDMs than in RAW264.7 macrophages. Thus, our results provide substantial new insights into macrophage biology and will likely necessitate a greater focus on studies examining BMDM activation.

Conclusively, using global profiling with a novel bioinformatics approach, we have described herein new molecules and signatures associated with different stages of the macrophage activation network. Our genome-wide, integrative analysis has revealed the integration of signaling crosstalk among IFN-γ and TLR4 at the level of the transcriptome in association with changes in related TFs and TcoFs. In addition, our data support the presence of transcriptional differences between BMDMs and RAW264.7 macrophages and between IL-4 and IL-13 M2 macrophage activation. Our findings thus provide novel insights into the dynamic regulation of inflammatory gene expression, potentially elucidating novel tools, and targets by which transcriptome regulators modulate macrophage activation and cytokine production.

## Accession Numbers

RNA-seq datasets supporting the results of this article are registered in the GEO database. The accession numbers are SRX897006, SRX897010, and GSE103958.

## Ethics Statement

All experimental protocols were conducted in accordance with Institutional Animal Care and Use Committee (IACUC) guidelines and approved by the IACUC committee of Hanyang University (HY-IACUC-2017-0004).

## Author Contributions

AD conceived and led the project. AD wrote the manuscript. AD, CY, SA, SK, SYK, and KJ performed all of the experiments and data collection. AD, YL, and YC performed the bioinformatics and transcriptional data analyses. AD provided critical revision of the manuscript for important intellectual content. AD, YC supervised the study. All of the authors were involved in proofreading the manuscript. All of the authors read and approved the final manuscript.

## Conflict of Interest Statement

The authors declare no conflicts of interest with respect to the financial, research, authorship, and/or publication of this article.

## Footnotes

^1^http://genome.ucsc.edu/.

^2^http://software.broadinstitute.org/gsea/msigdb/index.jsp.

^3^http://www.ingenuity.com.
