# Supplementary material for: High-Resolution Mapping and Dynamics of the Transcriptome, Transcription Factors, and Transcription Co-Factor Networks in Classically and Alternatively Activated Macrophages
Source: Front Immunol. 2018 Jan 18;9:22. doi: 10.3389/fimmu.2018.00022 (PMC5778122; doi:10.3389/fimmu.2018.00022)
Supplement: Supplementary file 1 [file Presentation_1.ZIP › Supplementary Figure S1, S2, S3, S4, S5, S6 and Table S1, S2.docx]

**Supplementary Figures**

**
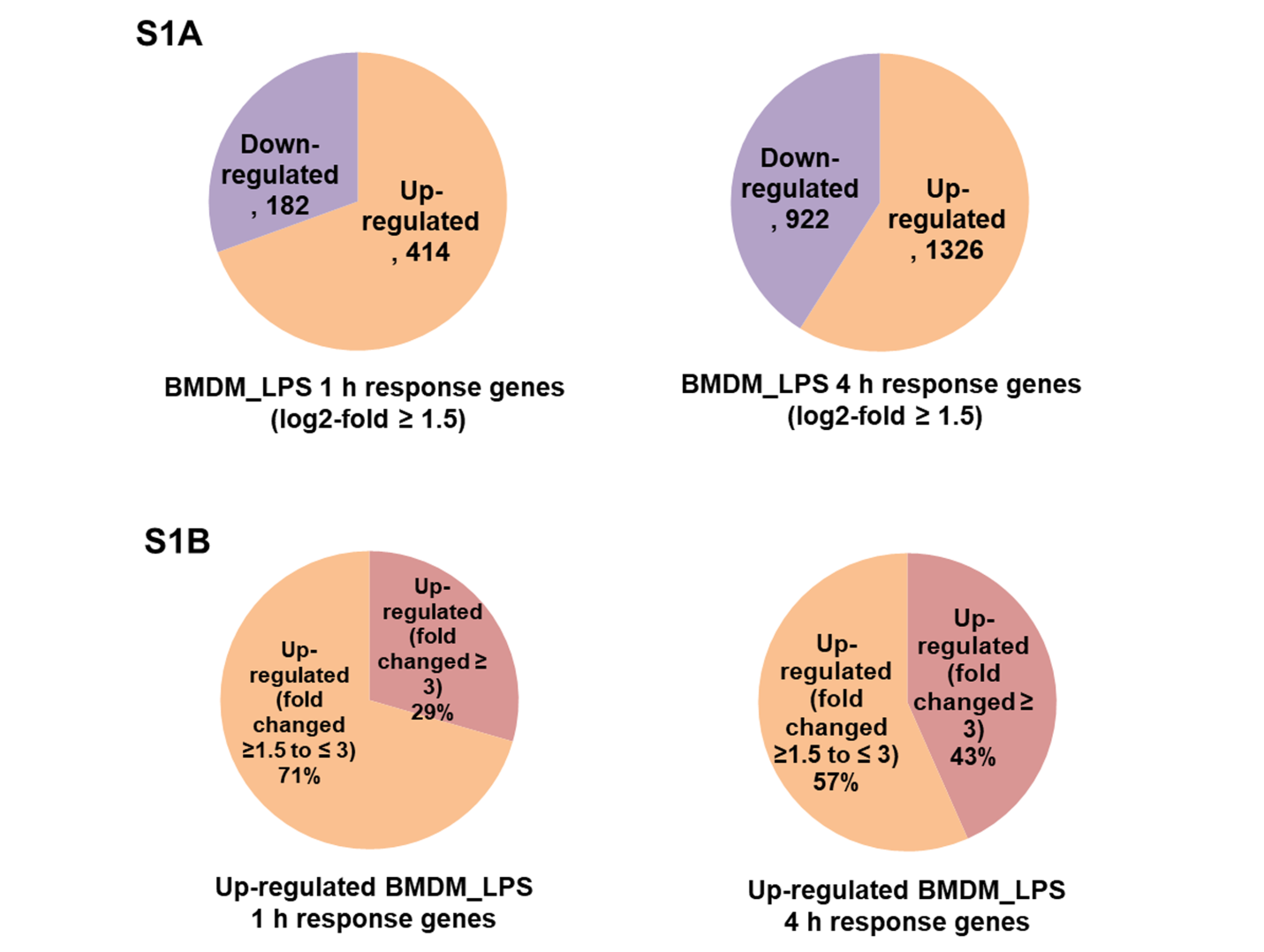
**

**Figure S1 Number of genes differentially expressed after LPS treatment in BMDMs. (S1A)** Pie chart displaying the number of up or down-regulated genes at 1 and 4 h after LPS stimulation in BMDMs. **(S1B)** Pie chart displaying the number of up-regulated genes (log_2-_fold change ≥ 3.0 and ≥ 1.5) at 1 and 4 h after LPS stimulation in BMDMs.

**
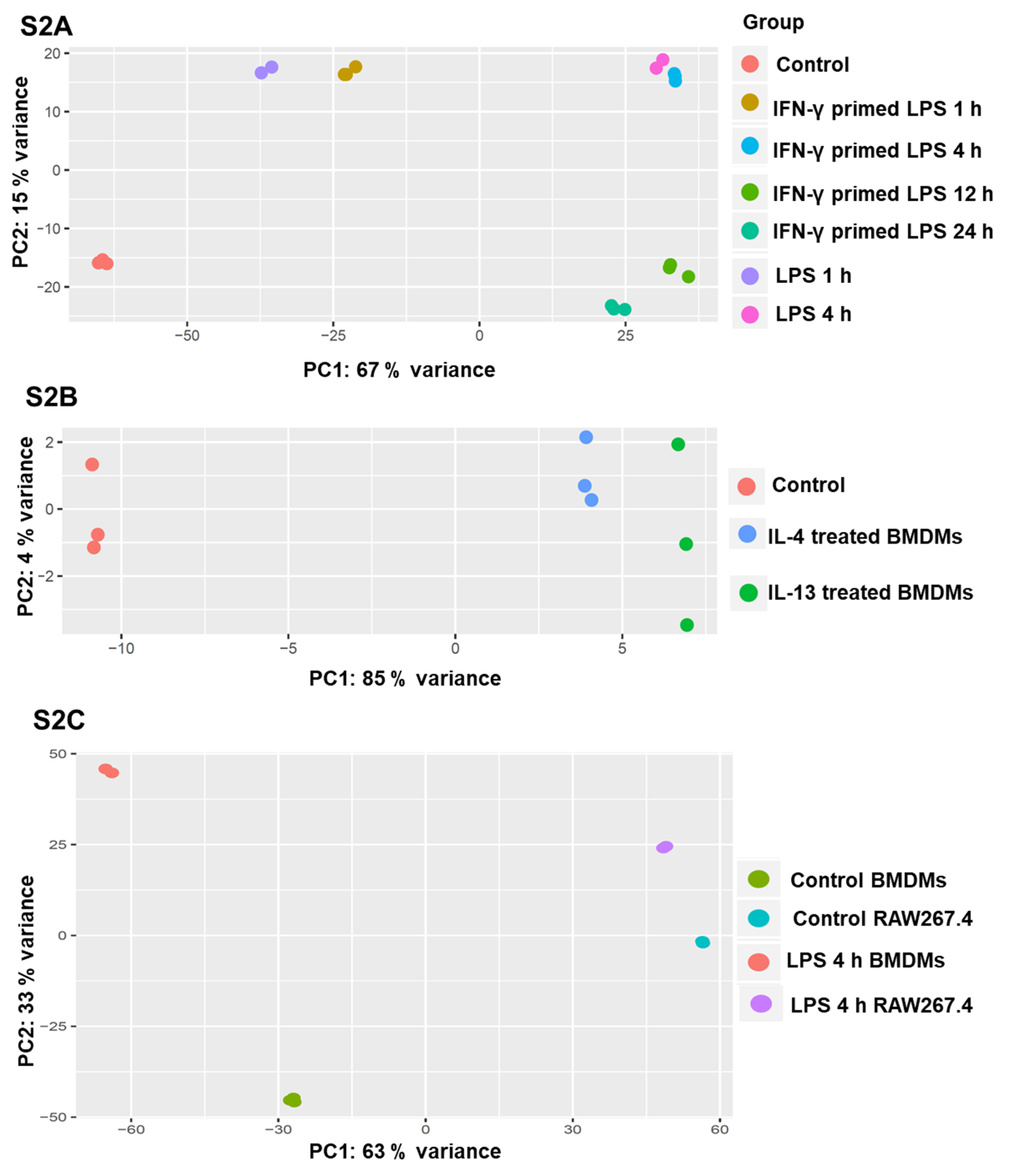
**

**Figure S2 PCA of data for different macrophage subtypes. (S2A)** The first principal component (PC1) separates untreated, IFN-γ-primed LPS-stimulated (1 h, 4 h, 12 h, and 24 h) and only LPS-treated (1 h, and 4 h) samples, while the second principal component (PC2) separates biological replicates of the same population in BMDMs. **(S2B)** The first PC1 separates untreated, IL-4-treated and IL-13-treated samples, while the second principal component (PC2) separates biological replicates of the same population in BMDMs. **(S2C)** The first PC1 separates untreated BMDMs, untreated RAW264.7 macrophages, LPS-treated BMDMs and LPS-treated RAW264.7 macrophages, while the second principal component (PC2) separates biological replicates of the same population in BMDMs and RAW264.7 macrophages.


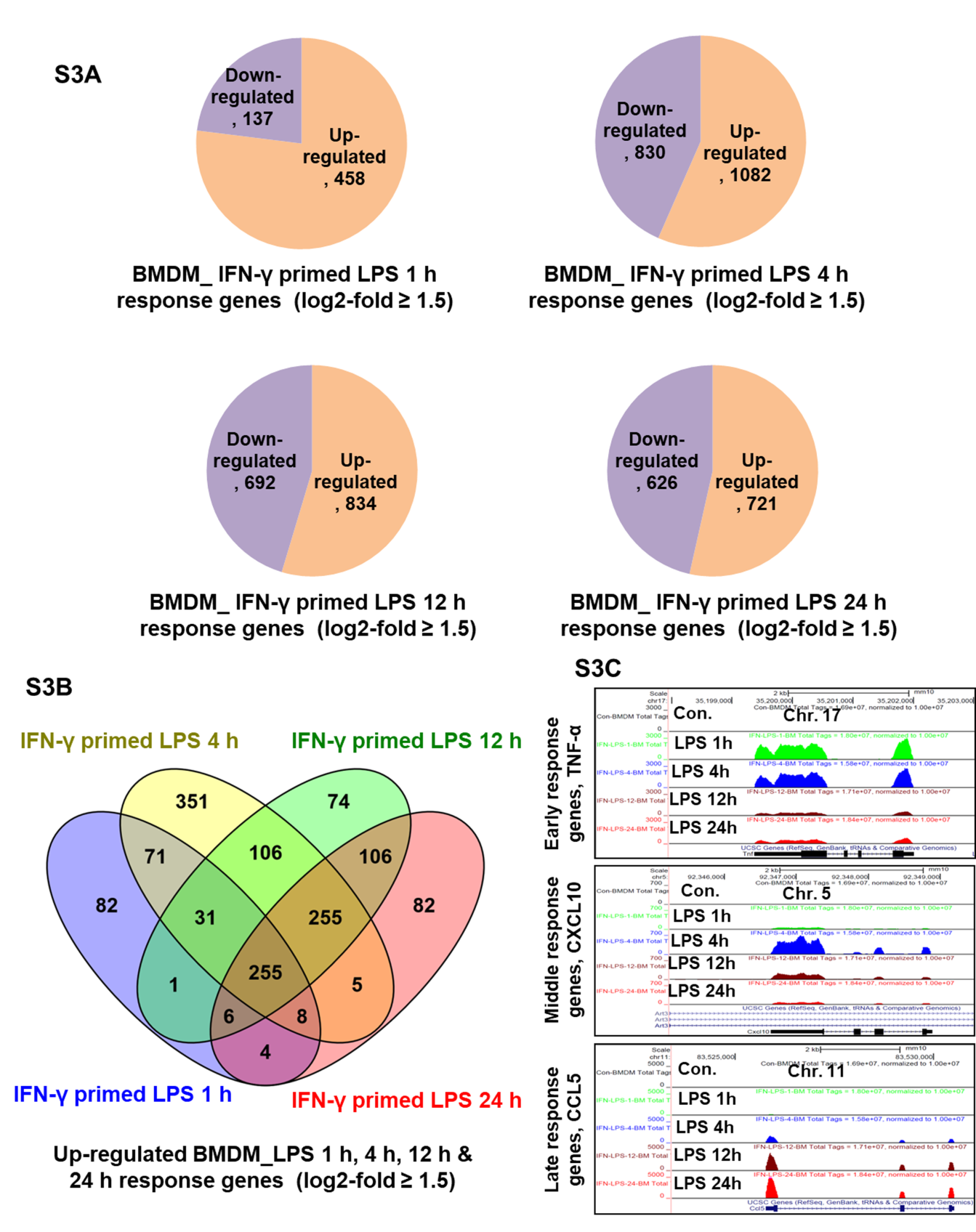


**Figure S3 Number of genes differentially expressed after IFN-γ-primed LPS-induced BMDMs. (S3A)** Pie chart displaying the number of up or down-regulated genes (*P* ≤ 0.01, and log_2-_fold change ≥ 1.5) at 1, 4, 12, and 24 h after IFN-γ-primed LPS stimulation in BMDMs. **(S3B)** Gene expression profile of the detected genes at 1, 4, 12, and 24 h after IFN-γ-primed LPS stimulation in BMDMs. Venn diagram depicting unique and intersecting genes at different time points. **(S3C)** UCSC Browser images representing the normalized RNA-seq read density in early (TNF-α), middle (CXCL10), and late (CCL5) up-regulated positive regulators of inflammatory genes in IFN-γ-primed LPS-stimulated BMDMs.

**
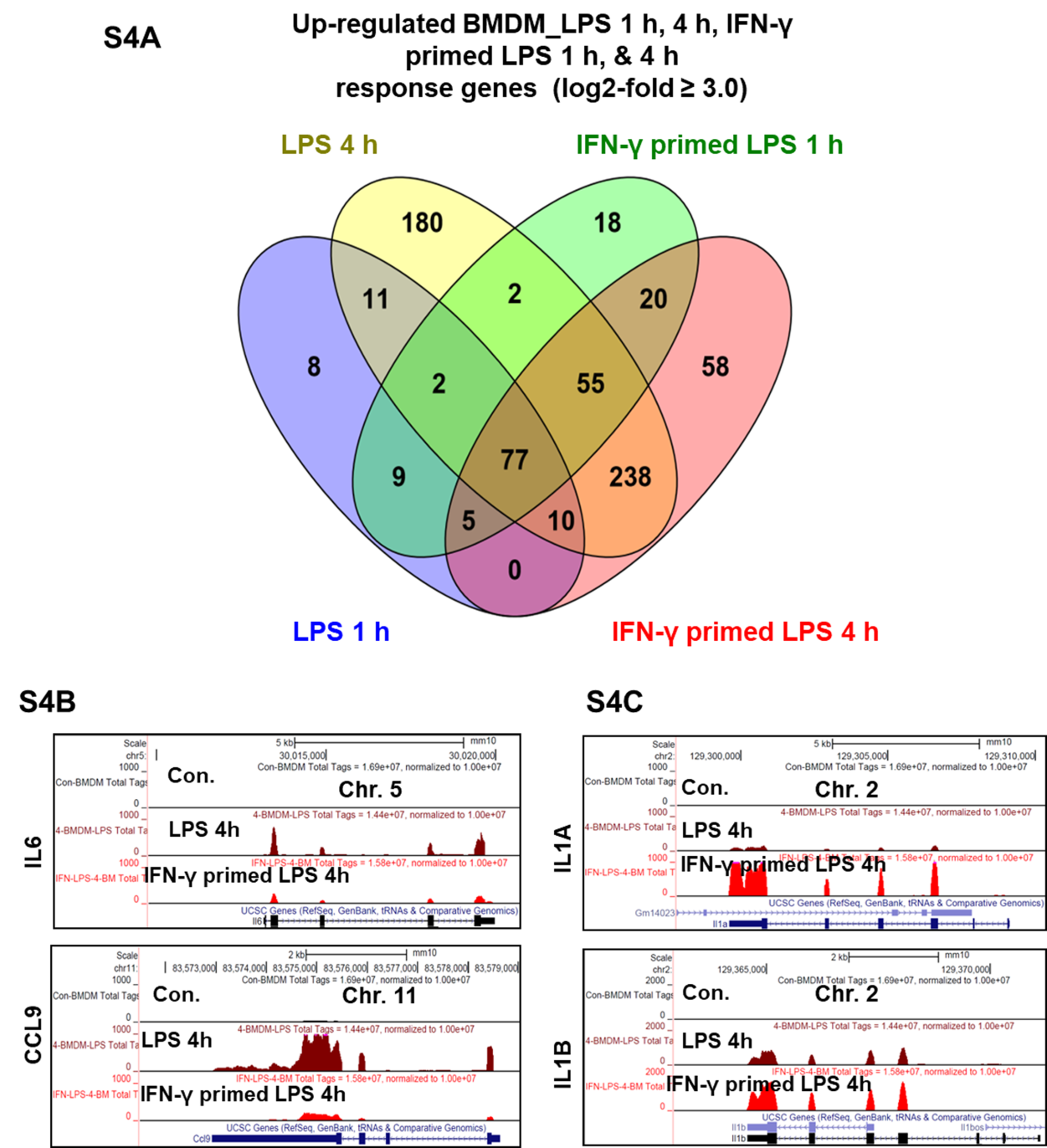
**

**Figure S4 Number of genes differentially expressed after only LPS treatment and IFN-γ-primed LPS-induced BMDMs. (S4A)** The Venn diagrams depict the overlapping genes between only LPS-inducible and IFN-γ-primed LPS-inducible BMDMs. **(S4B, C)** UCSC Browser images representing the normalized RNA-seq read density of inflammatory genes that were either down or up-regulated in IFN-γ-primed LPS-inducible BMDMs compared with only LPS-inducible BMDMs, respectively.

**
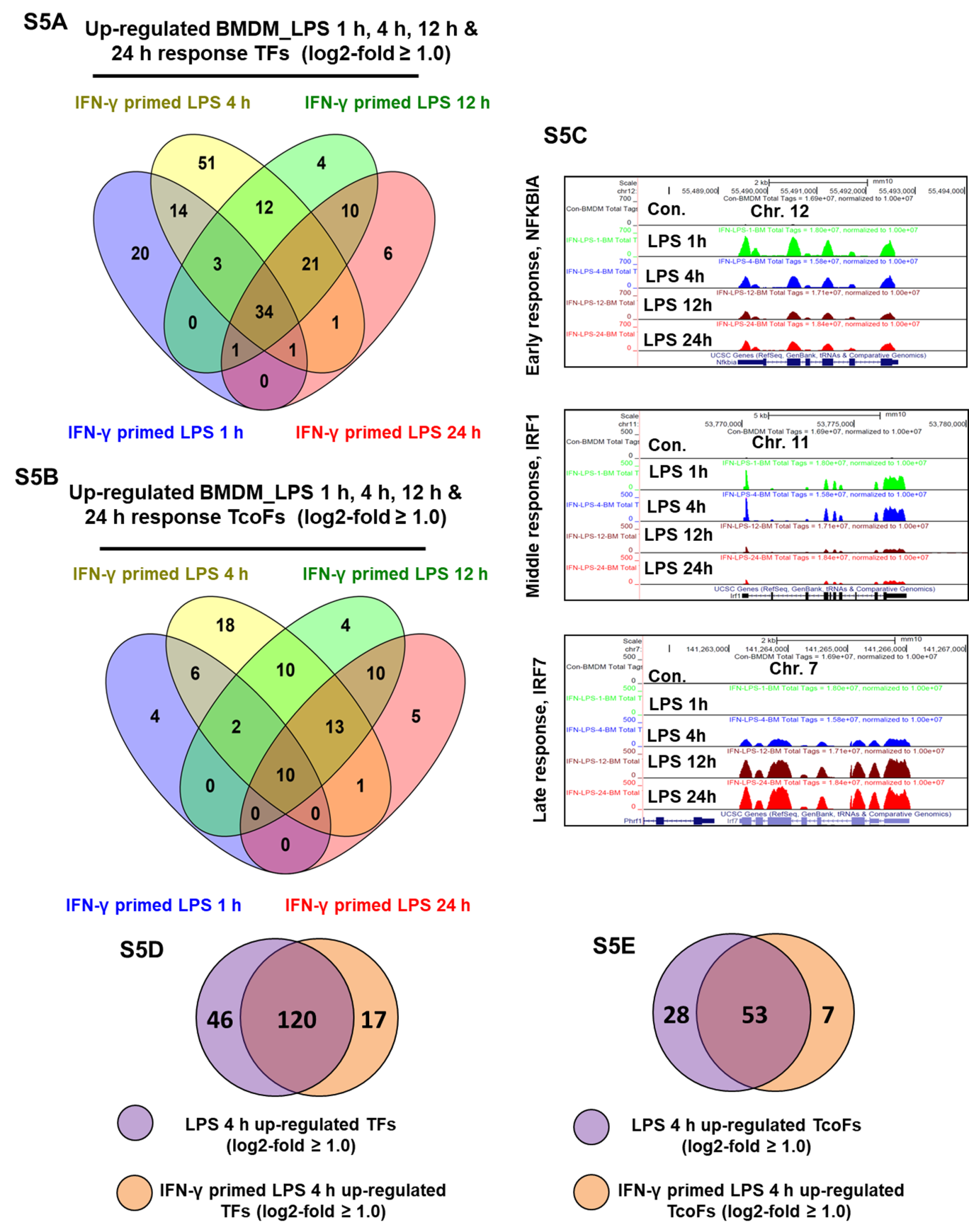
**

**Figure S5 Number of TFs and TcoFs differentially expressed after only LPS treatment and IFN-γ-primed LPS-induced BMDMs. (S5A, B)** TFs and TcoFs detected at 1, 4, 12, and 24 h after IFN-γ-primed LPS stimulation in BMDMs. Venn diagram depicting unique and intersecting TFs and TcoFs among the different time points. Venn diagram depicting unique and intersecting TFs and TcoFs among the different time points, respectively. **(S5C)** UCSC Browser images representing the normalized RNA-seq read density in early (NFkBIA), middle (IRF1), and late (IRF7) up-regulated TFs in IFN-γ-primed LPS-inducible BMDMs. **(S5D, E)** The area of overlap indicates the number of unique or shared up-regulated TFs and TcoFs between only LPS-inducible and IFN-γ-primed LPS-inducible BMDMs, respectively.


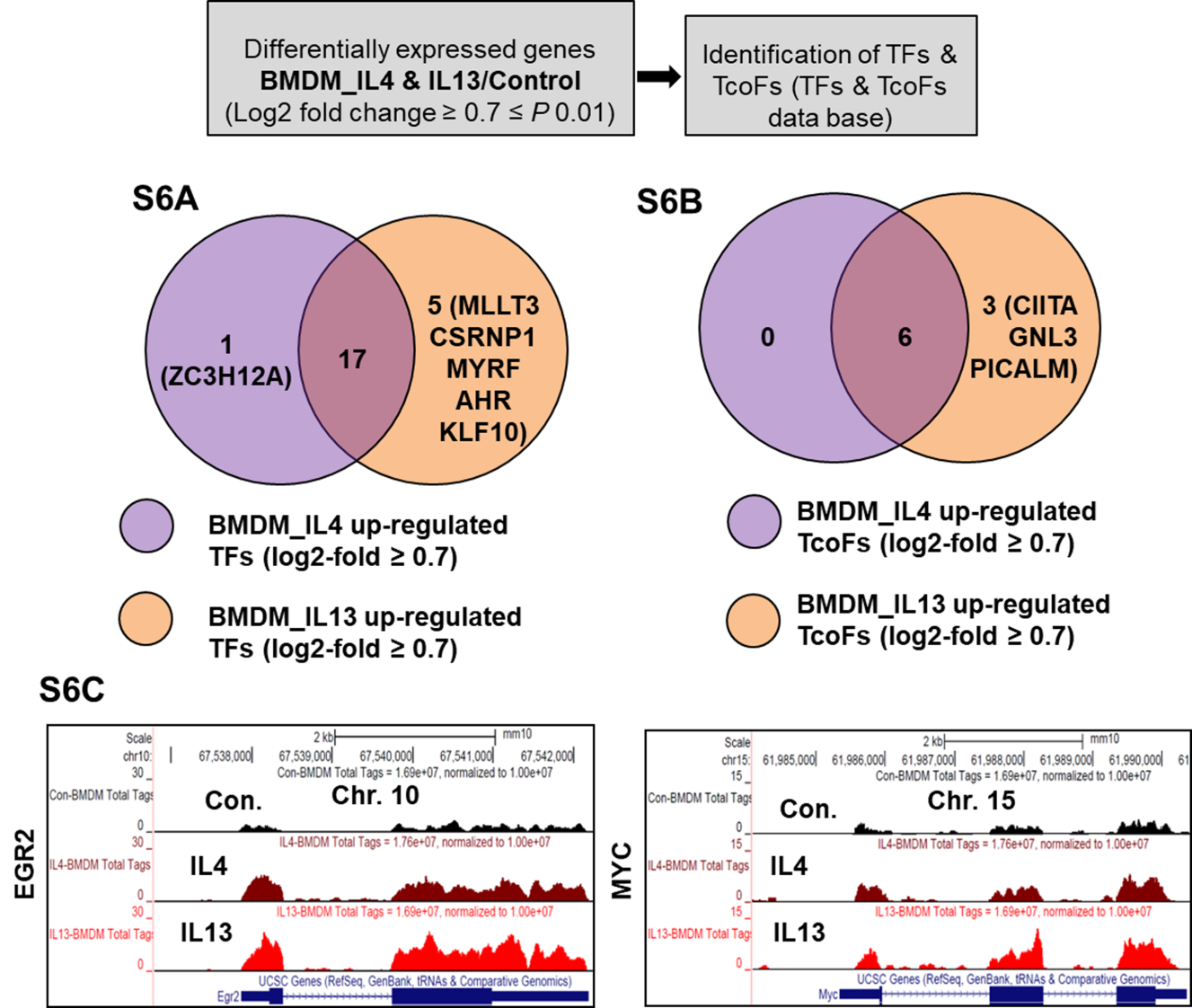


**Figure S6 Number of TFs and TcoFs differentially expressed after IL-4 and IL-13-treated BMDMs. (S6A, B)** Detected TFs and TcoFs in IL-4 and IL-13-treated BMDMs. Venn diagram depicting unique and intersecting TFs and TcoFs in IL-4 and IL-13-treated BMDMs, respectively. **(S6C)** UCSC Browser images representing the normalized RNA-seq read density in commonly expressed TFs between IL-4 and IL-13-treated BMDMs.

**Table S1: List of primers used in qRT-PCR studies.**

| Gene | Forward (5' -> 3') | Reverse (5' -> 3') |
| --- | --- | --- |
| *TNF-α* | CAGGCGGTGCCTATGTCTC | CGATCACCCCGAAGTTCAGTAG |
| *CXCL10* | TGCTGGGTCTGAGTGGGACT | CCCTATGGCCCTCATTCTCAC |
| *CCL5* | TTTGCCTACCTCTCCCTCG | CGACTGCAAGATTGGAGCACT |
| *IL6* | TAGTCCTTCCTACCCCAATTTCC | TTGGTCCTTAGCCACTCCTTC |
| *CCL9* | CCCTCTCCTTCCTCATTCTTACA | AGTCTTGAAAGCCCATGTGAAA |
| *IL1A* | TCTATGATGCAAGCTATGGCTCA | CGGCTCTCCTTGAAGGTGA |
| *IL1B* | GAAATGCCACCTTTTGACAGTG | CTGGATGCTCTCATCAGGACA |
| *NFKBIA* | TGAAGGACGAGGAGTACGAGC | TGCAGGAACGAGTCTCCGT |
| *IRF1* | ATG CCA ATCACTCGAATGCG | TTGTATCGGCCTGTGTGAATG |
| *IRF7* | GCGTACCCTGGAAGCATTTC | GCACAGCGGAAGTTGGTCT |
| *ARG1* | CTCCAAGCCAAAGTCCTTAGAG | GGAGCTGTCATTAGGGACATCA |
| *MRC1* | CTCTGTTCAGCTATTGGACGC | TGGCACTCCCAAACATAATTTGA |
| *EGR2* | CAGGAGTGACGAAAGGAAGC | GAAGACTGGGCAGATGGAGG |
| *MYC* | TGAGCCCCTAGTGCTGCAT | AGCCCGACTCCGACCTCTT |
| *IFIT1* | GCCTATCGCCAAGATTTAGATGA | TTCTGGATTTAACCGGACAGC |
| *GAPDH* | TGCGACTTCAACAGCAACTC | CTTGCTCAGTGTCCTTGCTG |

**Table S2: List of primers used in ChIP-PCR studies**

| Gene symbol | Forward Sequence (5' -> 3') | Reverse Sequence (5' -> 3') |
| --- | --- | --- |
| *TNF-α* | CCCCAGATTGCCACAGAATC | CCAGTGAGTGAAAGGGACAG |
| *CCL2* | AGCCAACTCTCACTGAAGCC | TCAAGCAGGAGGAGGGATCT |
| *CCL7* | GGCCAGCCTCACATTACACT | CTCTGCTCTTCTGGCAGCTC |
| *CXCL2* | CCAACCACCAGGCTACAGG | GCGTCACACTCAAGCTCTG |
| *CXCL10* | TCAGAACAGAAGCCGGAAGT | TCCTTGACCCTGTAACCACAC |
| *CHIL3* | CATTTGCCCTGCCTTTGG | TCTTTCATGGATATTGATTTCCTAAGAG |
| *ARG1* | TGAACAGGCTGTATTAGCCAACA | AGCACCCTCAACCCAAAGTG |
